# BCL-xL as a therapeutic target in cetuximab-refractory colorectal cancer

**DOI:** 10.1038/s41419-026-08434-5

**Published:** 2026-01-31

**Authors:** Stella Asmanidou, Julia Thiel, Thomas L. Ekstrom, Julia Schueler, Eva Oswald, Patrick Metzger, Andreas C. Blaumeiser, Melanie Boerries, Lisa-Marie Wiebl, Ronja Schiffler, Raluca Tamas, Frank Essmann, Meng Dong, Steven A. Johnsen, Roland E. Kontermann, Monilola A. Olayioye

**Affiliations:** 1 University of Stuttgart, Institute of Cell Biology and Immunology, Stuttgart, Germany; 2 Dr. Margarete Fischer-Bosch Institute of Clinical Pharmacology and University of Tübingen, Stuttgart, Germany; 3 Robert Bosch Center for Tumor Diseases, Stuttgart, Germany; 4 Mayo Clinic Graduate School of Biomedical Sciences, Mayo Clinic, Rochester, MN USA; 5 Charles River Laboratories Germany GmbH, Am Flughafen 12-14, 79108 Freiburg, Germany; 6 Institute of Medical Bioinformatics and Systems Medicine, Medical Center-University of Freiburg, Faculty of Medicine, University of Freiburg, Freiburg, Germany; 7 German Cancer Consortium (DKTK), partner site Freiburg, a partnership between DKFZ and Medical Center-University of Freiburg, Freiburg, Germany; 8 University of Tübingen, Tübingen, Germany; 9 University of Stuttgart, Stuttgart Research Center Systems Biology, Stuttgart, Germany

**Keywords:** Colorectal cancer, Cell death

## Abstract

Despite recent medical advances, colorectal cancer (CRC) remains the second-leading cause of cancer-related death worldwide. For patients with KRAS wild-type metastatic CRC, the monoclonal antibody cetuximab, which targets the epidermal growth factor receptor (EGFR), is an approved treatment option. However, therapeutic success is often limited by the emergence of drug-resistant cancer cell populations within a few months. Therefore, alternative strategies to effectively target cetuximab-refractory CRC are urgently needed. Here, we sought to identify second-line therapeutic strategies using a CRC cell line with acquired cetuximab resistance as a model. Transcriptomic profiling of the resistant cells identified the apoptosis pathway as a potential therapeutic target, which was supported by their increased susceptibility to BH3-mimetics targeting the anti-apoptotic proteins MCL-1 and BCL-xL under both 2D and 3D culture conditions. These findings were validated in organotypic CRC slice cultures generated from cetuximab-resistant patient-derived xenografts (PDXs). Multiplex immunofluorescence staining demonstrated that BCL-xL inhibition effectively triggered apoptosis in heterogeneous PDX tumor slice models, including models harboring oncogenic BRAF mutations. Our findings suggest that cetuximab-resistant CRC retains apoptotic competence, and that BCL-xL inhibition serves as a robust alternative therapeutic strategy that is largely independent of the tumor mutational profile.

## Introduction

Colorectal cancer (CRC) is one of the leading causes of cancer-related deaths worldwide. Although early disease stages are curable with surgery and chemotherapy, around 20% of the patients already suffer from metastasis at the time of diagnosis and another 25% are likely to develop it at later disease stages [1]. In this advanced disease stage, patient survival can be prolonged by the combined treatment with chemotherapeutic agents and the monoclonal antibody cetuximab, which targets the epidermal growth factor receptor (EGFR, ErbB1/HER1; [2]). Despite the initial efficacy of cetuximab against KRAS wild-type CRC, acquired resistance to the drug within 3–12 months is a clinical challenge in 65–70% of patients, resulting in cancer relapse and limited treatment options [3, 4].

Cetuximab resistance can be acquired through compensatory activation of alternative receptor tyrosine kinases or genetic alterations that constitutively activate downstream signaling cascades, such as the PI3K/mTOR and MAPK pathways [5, 6]. Frequently altered downstream effectors are the RAS proto-oncogenes, including KRAS ( ~ 40%), NRAS ( ~ 4%) and HRAS ( < 2%), causing primary and secondary resistance through constitutive MAPK pathway activation [6, 7]. Using the CRC cell line LIM1215 to establish a cetuximab-resistant model, previous findings demonstrated re-sensitization of KRAS-mutant clones to cetuximab by dual pharmacological MEK inhibition [8]. However, the therapeutic response to MEK inhibitors is often short-lived, as CRC is marked by a diverse array of genetic and non-genetic alterations within subclonal lineages that promote resistance, finally leading to treatment failure of these targeted approaches [2, 9].

This supports the need for therapeutic strategies targeting core survival mechanisms, for example by BH3-mimetics, which directly antagonize anti-apoptotic BCL-2 proteins to induce cell death. To date, the BCL-2 inhibitor venetoclax is approved for the treatment of hematologic diseases [10]. Although the potential of BH3-mimetics in solid tumors remains under investigation, pre-clinical studies have identified the BCL-2 family members MCL-1 and BCL-xL as promising drug targets in CRC, particularly in the context of chemoresistance [11, 12]. BH3-mimetics imitate the function of BH3-only proteins and directly bind to anti-apoptotic proteins, thereby promoting the activation of BAX and BAK. Active BAX and BAK convert to oligomers, leading to the permeabilization of the mitochondrial outer membrane and activation of caspases [13]. Of note, in CRC cell lines and organoids, cell death induction by BH3-mimetics was reported to occur independently of mutations in oncogenic drivers or the consensus molecular subtype [14, 15]. In addition, several reports have described enhanced efficacy of the BH3-mimetics when combined with chemotherapy or targeted therapies [10].

Here, we generated a CRC cell line model with an acquired cetuximab resistance. Transcriptome analysis identified the apoptosis pathway as an actionable target in the resistant cells, translating into increased susceptibility to cell death induction by BH3-mimetics under both 2D and 3D culture conditions. These findings were validated ex vivo using cetuximab-resistant tumor tissues from patient-derived xenografts, which preserve key characteristics of the original tumor and reflect patient heterogeneity [16]. Using this slice culture system, we provide evidence that BCL-xL inhibition constitutes an effective death-inducing therapeutic strategy for CRC tumors that fail to respond to cetuximab.

## Results

### Generation and characterization of a cetuximab-resistant CRC cell line model

**Fig. 1:**
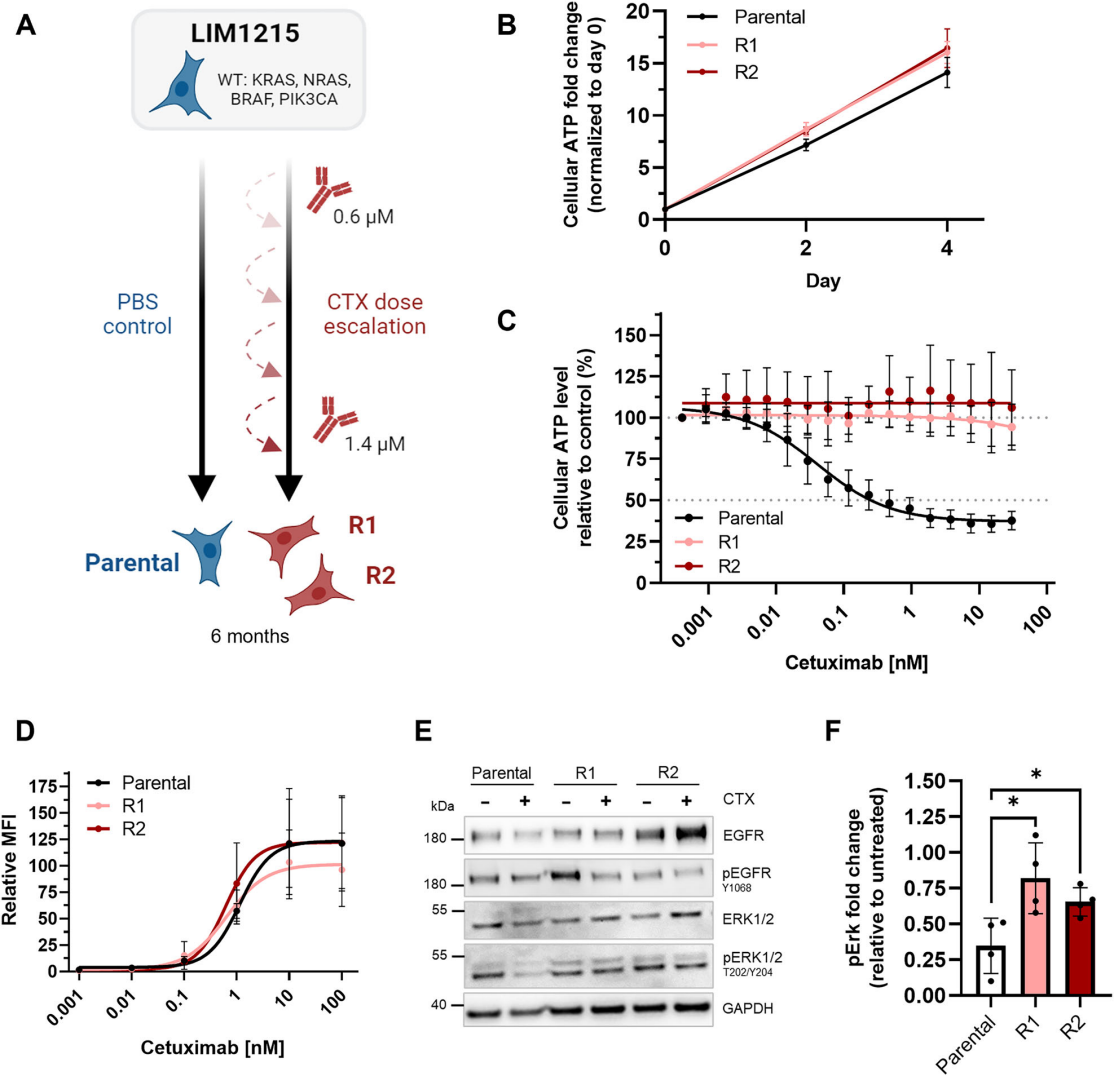
Characterization of the acquired cetuximab-resistant cell line model. **A** LIM1215 cells were treated for six months with a gradually increasing cetuximab concentration up to 1.4 µM. The two heterogenous cetuximab-resistant cell lines LIM1215-R1/R2 derived independently from the parental cell line. Over the treatment course of six months, the parental cell line was cultivated in parallel without cetuximab and served as a reference for subsequent experiments. **B** Cellular ATP levels of LIM1215 cell lines under basal culture conditions were determined using the CellTiter-Glo® 2.0 assay. Data were normalized to day 0 (mean ± SD; *n* = 3). **C** After long-term treatment, cetuximab was removed from the culture medium for 14 weeks and cells were re-treated for 5 days with increasing drug concentrations. Data shown as relative cellular ATP levels to control treatment. Cellular ATP levels were determined using the CellTiter-Glo® 2.0 assay (mean ± SD; *n* = 4). **D** Binding analysis of cetuximab to LIM1215 cell lines by flow cytometry analysis. Partial variability is observed due to biological heterogeneity among replicates, but overall trends are consistent across replicates. Data shown as mean fluorescence intensity (MFI, mean ± SD; *n* = 4). **E** Protein and phosphoprotein levels of LIM1215 cell lines after treatment with 100 nM cetuximab for 24 h. Whole cell lysates were analyzed by immunoblotting with the indicated antibodies and normalized to the loading control. One representative immunoblot image is shown from four biological replicates. **F** Quantification of pErk1/2 protein levels after exposure to cetuximab relative to the control treatment (PBS). pErk1/2 protein levels of each cell line were normalized to the loading control (mean ± SD; *n* = 4). Statistical significance was determined using a two-tailed unpaired t-test with Welch´s correction. *p < 0.05. CTX, cetuximab.

The limited availability of primary patient material underscores the need for appropriate in vitro models, particularly in cases of acquired drug resistance. Here, we generated an in vitro cetuximab-resistant cell line model by gradually exposing the initially sensitive, RAS wild-type CRC cell line LIM1215 to cetuximab over 6 months. Two independent resistant cell populations were created, referred to as LIM1215-R1 and LIM1215-R2 (Fig. 1A), with parental cells cultivated in parallel as controls (Fig. 1A; PBS control). In the absence of cetuximab, the proliferation rates of the resistant and parental cells were indistinguishable, indicating that the drug resistance does not impact the proliferative capacity of LIM1215-R1/R2 cells (Fig. 1B). To explore whether resistance was maintained after a drug holiday, LIM1215-R1/R2 cells were cultured without cetuximab for 14 weeks before re-exposure to the drug. As expected, 30 nM cetuximab reduced the viability of the parental cells by ~40% after 72 h, as indicated by ATP measurements, whereas LIM1215-R1/R2 cells remained insensitive even after the prolonged antibody removal (Fig. 1C).

Acquired cetuximab resistance can result from EGFR downregulation or mutation, leading to loss of target binding or inhibition [4]. However, flow cytometric analysis confirmed that cetuximab binding was preserved in LIM1215-R1/R2 cells (Fig. 1D), and total EGFR was not downregulated and presented with the correct molecular weight in western blot analysis (Fig. 1E). In addition, the ATP levels of LIM1215-R1/R2 cells remained unaffected by the EGFR-targeting antibody panitumumab which has overlapping but different EGFR binding epitopes compared to cetuximab (Fig. S1A), suggesting that drug resistance was not caused by EGFR extracellular domain mutations. Resistance to EGFR inhibitors is frequently mediated by compensatory signaling through the ErbB2/HER2 and ErbB3/HER3 family members and can involve autocrine production of HER ligands [17]. When compared to LIM1215-parental cells, total and surface HER2 and HER3 levels were not altered in LIM1215-R1/R2 cells (Fig. S1B, C). When blocking all three receptors using an antibody cocktail, ATP levels of LIM1215-R1/R2 cells were not reduced, indicating that resistance occurs independently of altered HER receptor signaling (Fig. S1D).

KRAS mutations in exon 2 and 3 are among the most frequent drivers of acquired cetuximab resistance [2, 6]. However, Sanger sequencing revealed that LIM1215-R1/R2 cells were wild-type for KRAS, with no indication of gene amplification or increased protein levels (Fig. S2A–C). Nevertheless, while cetuximab treatment reduced ERK1/2 phosphorylation (pERK1/2) in LIM1215-parental cells, pERK1/2 levels in LIM1215-R1/R2 cells remained comparably elevated (Fig. 1E, F), suggesting uncoupling of EGFR signaling and MAPK activation independently of KRAS mutations. This observation prompted us to target the MAPK pathway to suppress the viability of the LIM1215-R1/R2 cell lines.

### MEK1/2 inhibition does not restore cetuximab sensitivity of LIM1215-R1/R2 cells

**Fig. 2:**
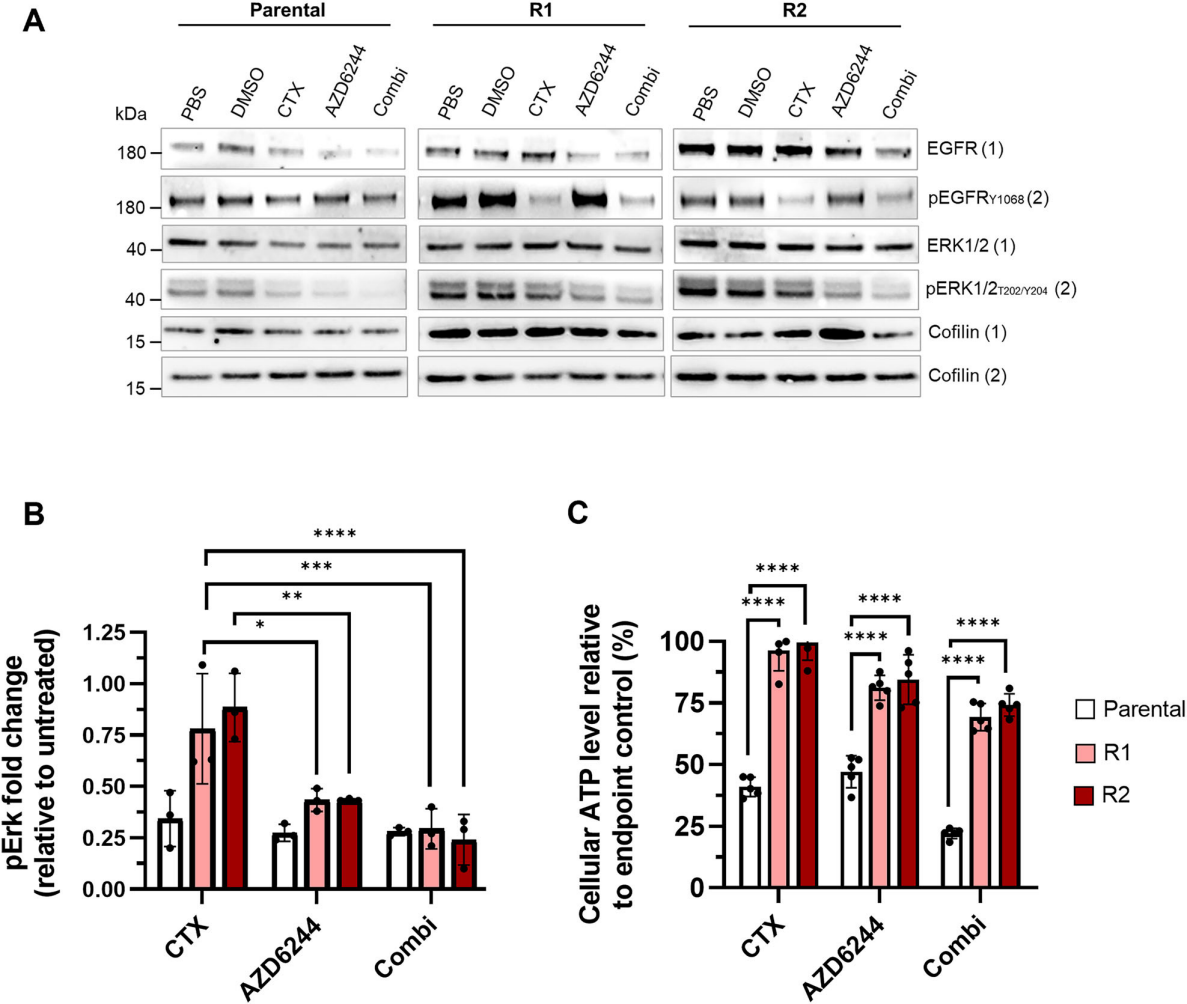
Cell viability of cetuximab-resistant LIM1215 cells upon dual EGFR and MEK1/2 blockade. **A** EGFR pathway targeting reflected in indicated protein and phosphoprotein levels by western blot analysis on whole-cell lysates. Cells were treated for 24 h with 50 nM cetuximab, 250 nM AZD6244 or a combination (combi) of both drugs. One representative immunoblot is shown from three biological replicates. Cofilin was included as a loading control. **B** Analysis of EGFR downstream signaling in response to dual EGFR and MEK inhibition. Western blots from three independent experiments were analyzed. pErk protein levels were normalized to GAPDH levels (mean ± SD). **C** Cellular ATP levels of LIM1215 in response to EGFR and MEK blockade after 3 days of treatment with 50 nM cetuximab, 250 nM AZD6244 or a combination of both drugs. ATP levels were determined using the CellTiter-Glo® 2.0 assay. Data shown as relative cellular ATP levels to endpoint control treatment (mean ± SD, *n* = 5). Statistical significance was determined using two-way ANOVA with Tukey post-hoc test. *p < 0.05, **p < 0.01, ***p < 0.001, ****p < 0.0001. CTX, cetuximab.

Constitutive ERK1/2 activation can be abrogated by MEK inhibitors such as AZD6244 which was shown to sensitize cancer cells to cetuximab [18, 19]. Indeed, after 24 h of treatment with AZD6244 ± cetuximab, pERK1/2 levels decreased across all three cell lines (Fig. 2A, B). As expected, the cellular ATP levels of LIM1215-parental cells were strongly decreased in response to AZD6244 ± cetuximab. Surprisingly, treatment with AZD6244 alone reduced the ATP levels of LIM1215-R1/R2 cells only slightly (81% and 85%, respectively). Even upon addition of cetuximab, the ATP levels of the resistant cells decreased only modestly to 69% and 74%, thus remaining significantly elevated compared to LIM1215-parental cells (Fig. 2C). This indicates that compensatory pathways other than the MAPK pathway are responsible for the observed cetuximab resistance of LIM1215-R1/R2 cells.

### Cetuximab-resistant LIM1215 cells are sensitive towards cell death-inducing agents

**Fig. 3:**
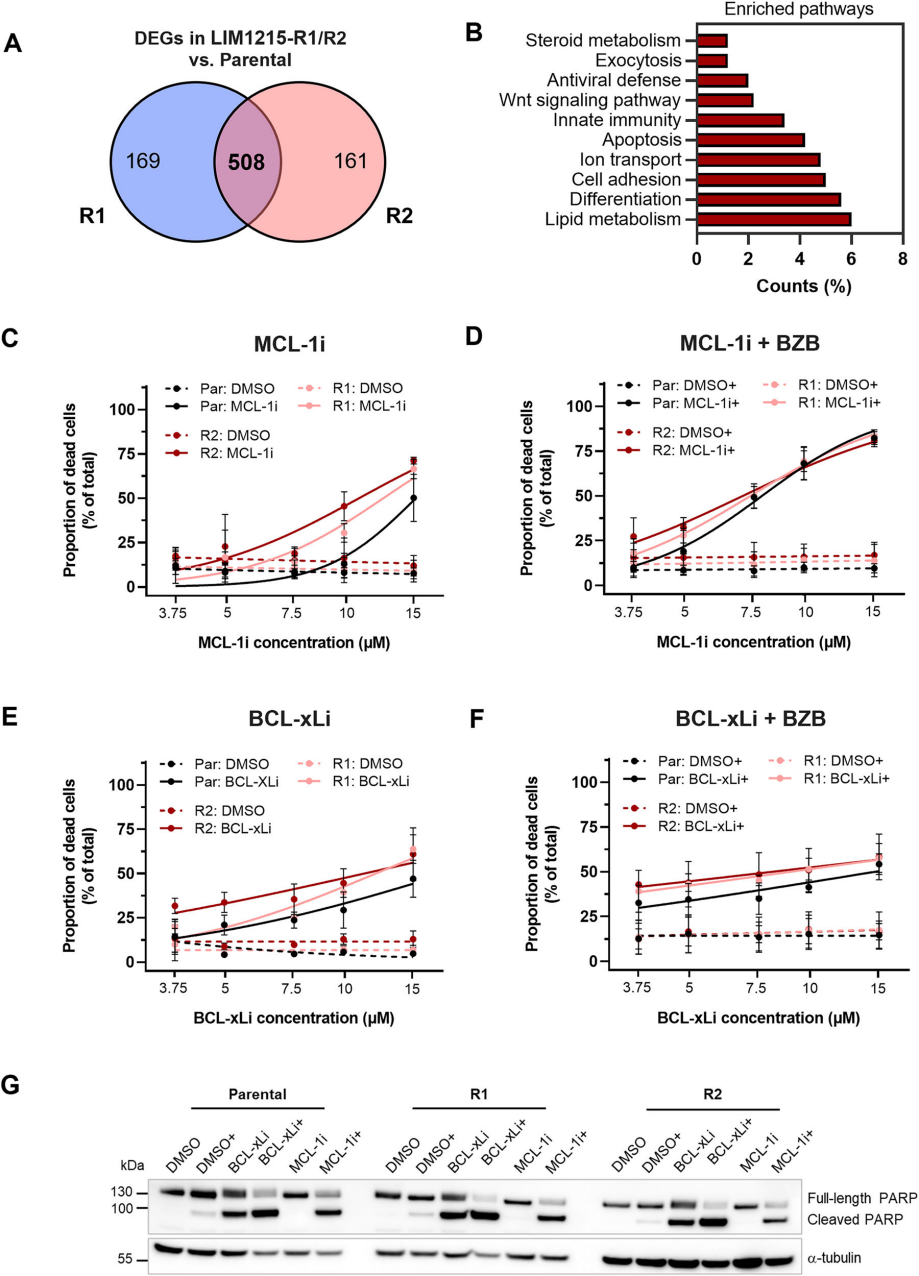
Cetuximab-resistant LIM1215 cell lines are sensitive towards cell death-inducing agents. **A** Transcriptome analysis by RNA sequencing revealed that 508 genes shared by the two resistant cell lines are differentially expressed relative to the control LIM1215-parental cell line. **B** Differentially expressed genes (DEGs) shared by both resistant cell lines (either up- or down-regulated) were analyzed for the generation of top annotation clusters by the DAVID Functional Annotation Clustering Tool. **C–F** Cell death analysis after 24 h of treatment with increasing BH3-mimetic drug concentrations (MCL-1i or BCL-xLi) ± 5 nM bortezomib (addition indicated with +). Cells were stained with Annexin V-GFP and propidium iodide, followed by flow cytometric analysis. The proportion of dead cells was determined by combining the proportion of Annexin V + /PI+ cells (double-positive) and Annexin V+ only cells (mean ± SD). **C** Monotherapy with MCL-1i (*n* = 4). **D** Dual treatment with MCL-1i and 5 nM bortezomib (MCL-1i + ; *n* = 3). **E** Monotherapy with BCL-xLi (*n* = 3). **F** Dual treatment with BCL-xLi and 5 nM bortezomib (BCL-xLi + ; *n* = 3). **G** Cleaved PARP protein levels after 24 h of treatment with 10 µM BH3-mimetics alone or in combination with 5 nM bortezomib (addition indicated with +). Whole-cell extracts were subjected to western blot analysis. α-tubulin was included as a loading control. Par parental, CTX cetuximab, BZB bortezomib, PI propidium iodide, α alpha.

To gain a comprehensive understanding of the molecular alterations in LIM1215-R1/R2 cells, bulk RNA sequencing analysis of parental and resistant LIM1215 cells was performed. Of note, the analysis of mutational status via short read sequencing has its limitations, especially when covering long transcripts. Nevertheless, it confirmed the KRAS wild-type status of both resistant cell lines, and interestingly revealed activating HRAS mutations in exon 2 (LIM1215-R1) and exon 3 (LIM1215-R2), respectively. These mutations were detected in a subpopulation of the resistant cell lines (data not shown), reflecting their mixed, non-clonal nature. Additionally, no activating mutations of oncogenic drivers such as NRAS and BRAF were detected (Table S1). Further analysis of the RNA sequencing data revealed extensive and reproducible transcriptomic alterations that were shared between LIM1215-R1/R2 cells when compared to the parental cell line (Fig. S3; Fig. S4). We identified 508 commonly differentially expressed genes (DEGs) in both resistant cell lines compared to the parental cell line (adjusted p-value ≤ 0.01 and log_2_ fold change ≥ 1.5; Fig. 3A). Analysis of top altered biological processes included lipid metabolism, differentiation, cell adhesion, ion transport, and apoptosis (Fig. 3B; Table S2 and S3). Given the central role of apoptosis as a hallmark of cancer and drug resistance, we further analyzed the expression of key apoptosis regulators at the protein level under basal conditions and upon cetuximab exposure. While BCL-2 could not be detected and MCL-1 was equally expressed in all three cell lines, BCL-xL was significantly increased in LIM1215-R2, and modestly elevated in LIM1215-R1 cells under basal conditions compared to LIM1215-parental cells (Fig. S5A, B). Of the pro-apoptotic proteins, NOXA, PUMA and BAX could not be reliably detected due to their low or absent expression. We observed slightly decreased BAK protein levels in the two resistant cell lines, and a modest increase of BID in LIM1215-R2 cells, both under basal conditions. Upon cetuximab exposure, BIM levels increased in the parental and LIM1215-R2 cells, but remained unchanged in LIM1215-R1 cells (Fig. S5C–E).

Motivated by these observations, we next investigated the response of LIM1215-R1/R2 cells to cell death-inducing agents. Cell death induction can be achieved by BH3-mimetics directed at the anti-apoptotic proteins BCL-2, BCL-xL, and MCL-1. Based on the protein expression data, we focused on the latter two and selected the BH3-mimetics A-1155463 (BCL-xLi) and A-1210477 (MCL-1i) for further studies. Cell death analysis by flow cytometry following 24 h of treatment with the BH3-mimetics revealed sensitivity of all three cell lines to MCL-1 and BCL-xL inhibition (Fig. 3C–F). Of note, when compared to the parental cells, lower drug concentrations triggered cell death more effectively in the cetuximab-resistant cells (see 10 µM MCL-1i and 5-10 µM BCL-xLi, particularly in the case of LIM1215-R2; Fig. 3C, E). We furthermore explored the addition of a low concentration of the proteasome inhibitor bortezomib (5 nM), known to enhance the efficacy of BH3-mimetics by promoting the accumulation of pro-apoptotic proteins [20–22]. In the case of BCL-xLi, bortezomib addition only moderately increased the treatment response when compared to monotherapy (Fig. 3F). In contrast, bortezomib strongly increased the cell death response induced by 5 µM and 7.5 µM MCL-1i, approximately doubling the proportion of dead cells when compared to MCL-1i monotherapy (Fig. 3D). The induction of cell death was further verified by western blot analysis of cleaved PARP protein levels. While single BCL-xLi treatment effectively induced PARP cleavage across all cell lines, MCL-1i alone was insufficient and required the addition of bortezomib (Fig. 3G). Titration experiments further confirmed this enhanced apoptotic response of the LIM1215-R1/R2 cells in comparison to the parental cells (Fig. S6A, B).

Next, we investigated whether the cell death response was caspase-dependent by applying the pan-caspase inhibitor Q-VD-OPh. Upon exposure to 10 µM BCL-xLi ± bortezomib, flow cytometric analysis revealed a rescue from cell death by Q-VD-OPh in all cases, providing evidence for the engagement of caspases (Fig. S7A, B). By contrast, Q-VD-OPh only partially protected the cells from MCL-1i-induced cell death, in line with the low PARP protein cleavage (Fig. S7C; Fig. 3G). Notably, while Q-VD-OPh fully prevented cell death of the parental cells upon combined MCL-1i and bortezomib treatment, a significant proportion of the resistant cells underwent death despite caspase inhibition (Fig. S7D), suggesting the involvement of additional caspase-independent mechanisms triggering cell death.

We next subjected cells to combined BCL-xLi and MCL-1i treatment, which increased cell death over monotherapy only at a high concentration (5 µM) and revealed no synergy at lower doses. Notably, bortezomib with a low-dose of BCL-xLi (1.25 µM) already induced a strong cell death response, whereas the BH3-mimetic combination required the highest concentration to achieve similar effects (Fig. S8A, B).

Since the resistant cell lines harbored subpopulations with HRAS mutations, we assessed how pre-treatment with AZD6244 ± cetuximab affects their BH3-mimetic sensitivity. After seven days of treatment, cell death assays revealed a modest sensitivity decrease of LIM1215-R1/R2 cells to the single BH3-mimetics, but not of LIM1215-parental cells (Fig. S9A–C). In combination with bortezomib, LIM1215-parental cells gained resistance to MCL-1i, but not BCL-xLi, whereas LIM1215-R1/R2 cells were less sensitive BCL-xLi (Fig. S9D–F). Western blot analysis showed a significant decrease in HRAS and HRAS G12D expression in the LIM1215-R1 cell population by AZD6244, which was further enhanced by cetuximab addition, indicating that the mutant clones were suppressed by the pre-treatment (Fig. S10A–D). Notably, these pre-treated cells were still susceptible to cell death and retained apoptosis competence comparable to the parental cells.

### The combination of BH3-mimetic drugs with bortezomib reduces the viability of 3D spheroids

**Fig. 4:**
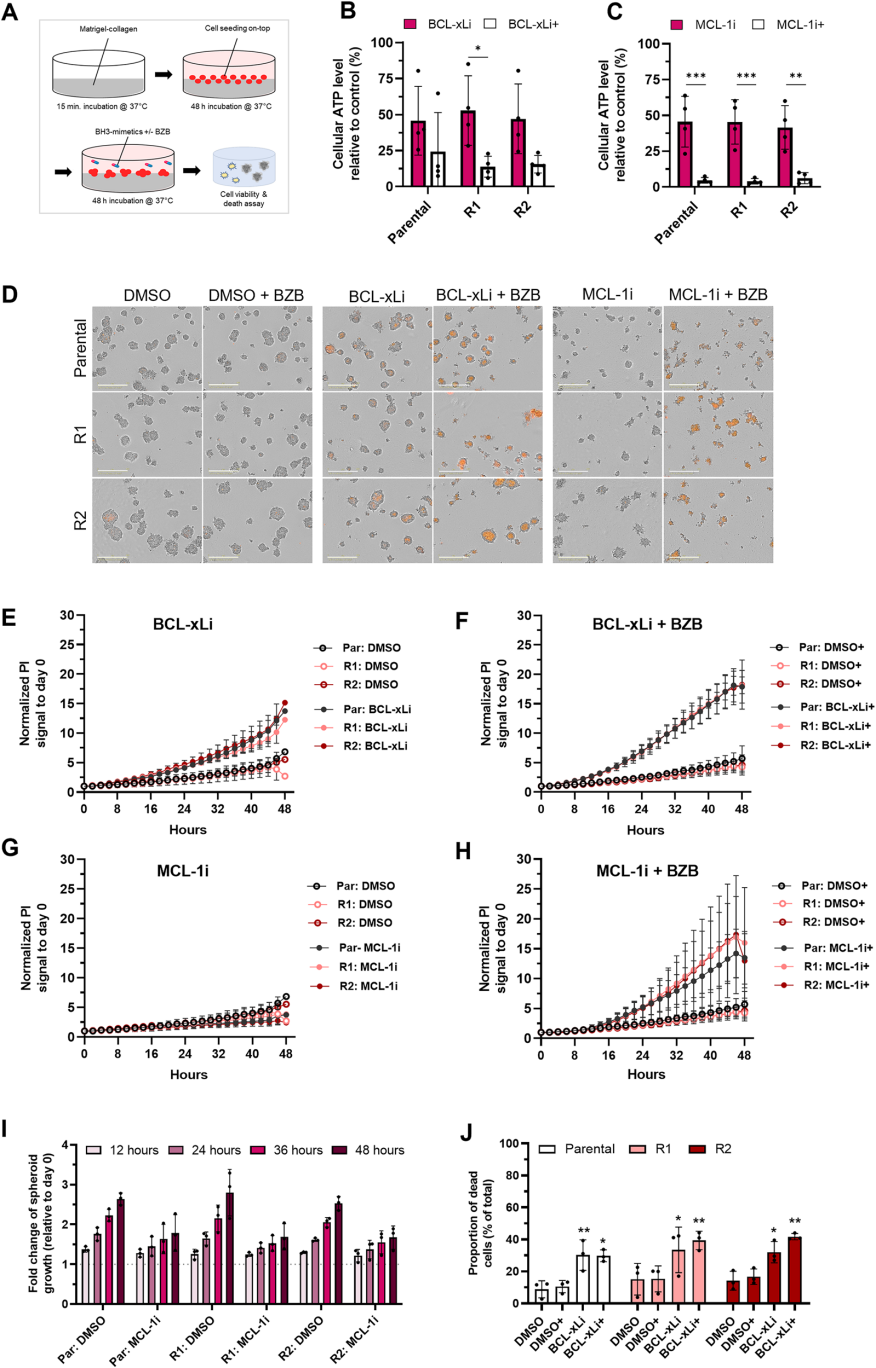
Treatment response of 3D-cultured LIM1215 cell lines to BH3-mimetic drugs. **A** Generation of 3D cell culture models on-top of a Matrigel-collagen bed. **B, C** Parental and resistant LIM1215 cell cultures were treated with 10 µM of BH3-mimetic drugs targeting **B** BCL-xL ± 5 nM bortezomib or (**C**) MCL-1 ± 5 nM bortezomib. Cellular ATP levels were determined using the CellTiter Glo® 3D assay. Data shown as relative ATP levels to control treatment (mean ± SD, *n* = 3). Statistical significance was determined using two-way ANOVA with Šidák post-hoc test. **D** Representative Incucyte images of 3D cultures stained with 0.5 µg/ml propidium iodide and treated with the aforementioned drug combinations. Scale bars: 400 µM. **E–H** Cell death analysis based on PI staining intensity in response to BH3-mimetic drug treatment by live-cell imaging using the Incucyte. 3D cultures were treated for 48 h with **E** 10 µM BCL-xLi alone or **F** in combination with 5 nM bortezomib (+), as well as with **G** 10 µM MCL-1i or **H** in combination with 5 nM bortezomib (+). Cell death response was quantified using the Incucyte analysis tool “All Brightfield Relative Total Red Integrated Intensity”, and data were normalized to day 0 (mean ± SD, *n* = 3). **I** Growth analysis of the 3D cultures in response to the BH3-mimetic MCL-1i by live-cell imaging using the Incucyte (mean ± SD, *n* = 3). **J** Cell death analysis of 3D cultures after 48 h of treatment with 10 µM BCL-xLi ± 5 nM bortezomib (addition indicated with +). Cells were stained with Annexin V-GFP and PI, followed by flow cytometric analysis. The proportion of dead cells was determined by combining the proportion of Annexin V + /PI+ cells (double-positive) and Annexin V+ only cells (mean ± SD, *n* = 3). Statistical significance was determined using two-way ANOVA with Tukey post-hoc test. *p < 0.05, **p < 0.01, ***p < 0.001. BZB bortezomib, par parental, PI propidium iodide.

To interrogate the therapeutic potential of the BH3-mimetics in an environment that resembles the in vivo setting, we investigated their efficacy in matrix-containing 3D cultures. Cells suspended in Matrigel were allowed to form spheroids, and treated with BH3-mimetics ± bortezomib for 48 h (Fig. 4A). Monotherapy with the BH3-mimetics decreased the cellular ATP levels by approximately 50%, and this suppression was amplified by low doses of bortezomib, with a particularly strong effect in the case of MCL-1i, leading to a cellular ATP level decrease of around 95% across the three cell lines (Fig. 4B, C). Furthermore, we monitored the drug response in real-time in the presence of propidium iodide (PI; Fig. 4D–H), using the integrated intensity of the PI signal as a proxy for the degree of cell death (see representative images for the 48-h time point; Fig. 4D). Consistent with the cell viability data (Fig. 4B), BCL-xLi induced cell death in all three cell lines, which was enhanced by bortezomib addition (Fig. 4E, F). The death response induced by BCL-xLi ± bortezomib was verified by flow cytometry analysis using Annexin/PI-staining (Fig. 4I). Interestingly, while treatment with MCL-1i reduced the viability, as indicated by cellular ATP and spheroid growth measurements (Fig. 4C, J), accumulation of the PI signal was only observed upon co-treatment with bortezomib (Fig. 4G, H), in line with the 2D assay data. Taken together, our findings demonstrate that despite acquired cetuximab resistance, LIM1215-R1/R2 cells remained sensitive to BH3-mimetics combined with bortezomib.

### BCL-xL inhibition causes cell death in cetuximab-resistant PDX tumor tissues

**Fig. 5:**
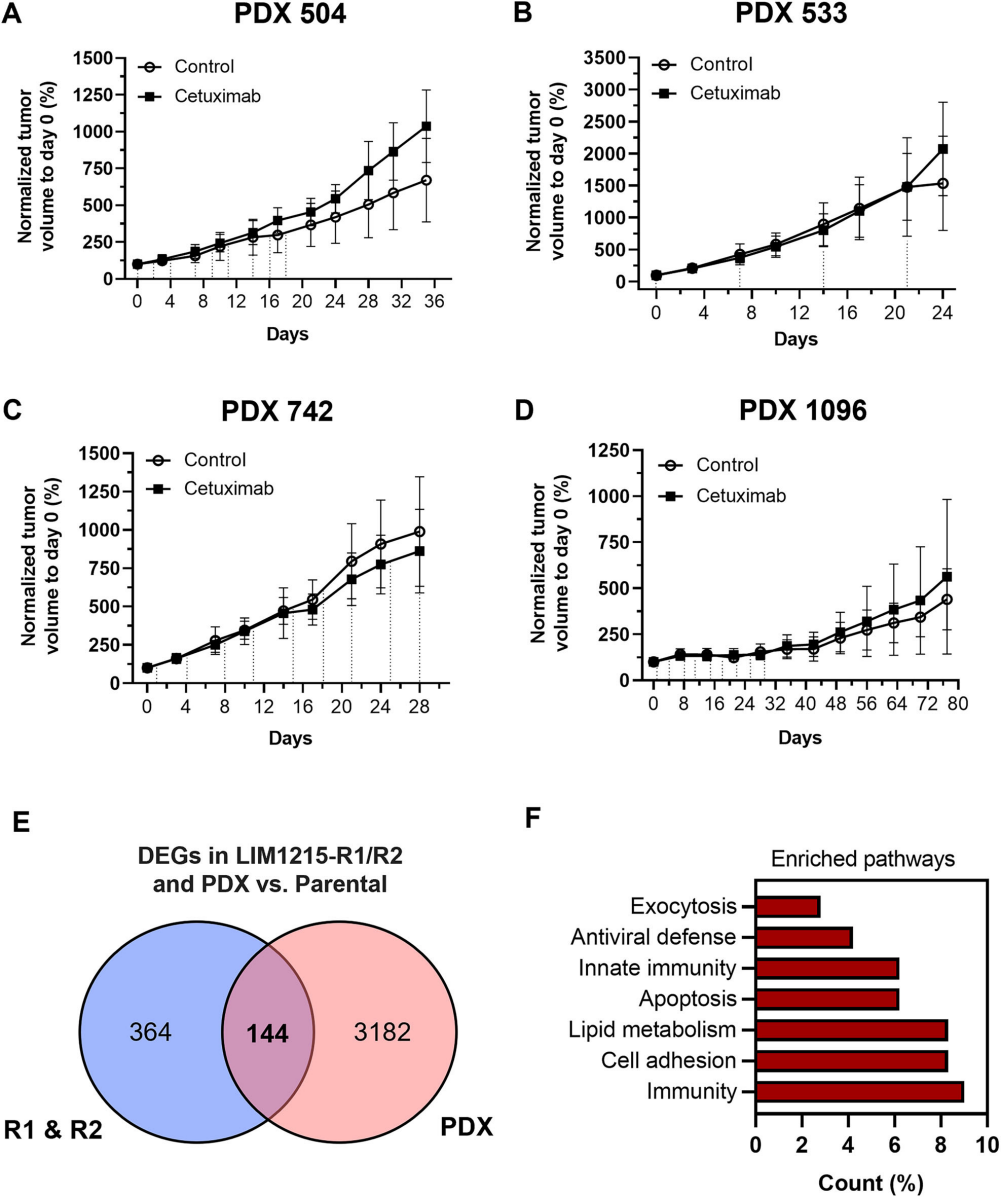
Transcriptome analysis of KRAS wild-type, cetuximab-resistant PDX-models reveals an altered apoptosis pathway. **A–D** Growth curve of subcutaneous patient-derived xenograft (PDX) fragments from cetuximab-resistant, KRAS wild-type PDX models 504, 533, 742, and 1096. Dashed lines indicate the intraperitoneal injection of cetuximab or control vehicle. All mice were randomly assigned to treatment groups. Data shown as normalized tumor volume to day 0. **A** Treatment of 7 mice harboring PDX model 504 with 20 mg/kg CTX or vehicle for 35 days. **B** Treatment of 10 mice harboring PDX model 504 with 25 mg/kg CTX or vehicle for up to 24 days. **C** Treatment of 10 mice harboring PDX model 742 with 50 mg/kg CTX or vehicle for up to 28 days. **D** Treatment of 10 mice harboring PDX model 742 with 30 mg/kg CTX or vehicle for up to 77 days. **E** Transcriptome analysis of DEGs shared between the four resistant PDX models and LIM1215-R1/R2 cells, using LIM1215 parental cells as reference. **F** Biological processes shared by cetuximab-resistant PDX models and LIM1215-R1/R2 cells determined by DAVID Functional Annotation Bioinformatics Microarray Analysis. CTX, cetuximab. Data shown as mean ± SD.

Finally, we validated the efficacy of the BH3-mimetics on patient-derived CRC xenograft tissues that retain microenvironmental features of the tumor and reflect patient heterogeneity (Table S4). We selected four cetuximab-resistant and KRAS wild-type CRC patient-derived xenograft (PDX) models, thereby matching the key characteristics of the LIM1215-R1/R2 cell lines. The additional mutational profile of the PDX models was determined by whole exome sequencing, revealing alterations in BRAF (activating V600E mutations in PDX 504 and 742), TP53 (missense mutations in all models) and PTEN (gene deletion in PDX 1096), but no hotspot mutations in HRAS, NRAS, PIK3CA, and EGFR (Table S5). Intrinsic drug resistance of these models was scored in vivo by subjecting mice harboring the PDX tissues to cetuximab treatment with doses of 20–50 mg/kg for 24–77 days, revealing negligible growth suppression by the antibody and resistance scores of 62–91% relative to the control vehicle (Fig. 5A–D; Fig. S11). To further identify shared molecular features between the PDX tumors and LIM1215-R1/R2 cells, we compared the DEGs of both model systems, using LIM1215-parental cells as a reference. LIM1215-R1/R2 cells shared 144 DEGs with the four selected PDX models (Fig. 5E), and the apoptosis pathway emerged as a hallmark of the resistant models, consistent with previous findings in LIM1215-R1/R2 cells (Fig. 5F). Given the enhanced cell death observed in 2D and 3D cell culture upon MCL-1i or BCL-xLi + bortezomib, we next aimed to validate these promising drug combinations in ex vivo PDX-derived tumor slice cultures.

**Fig. 6:**
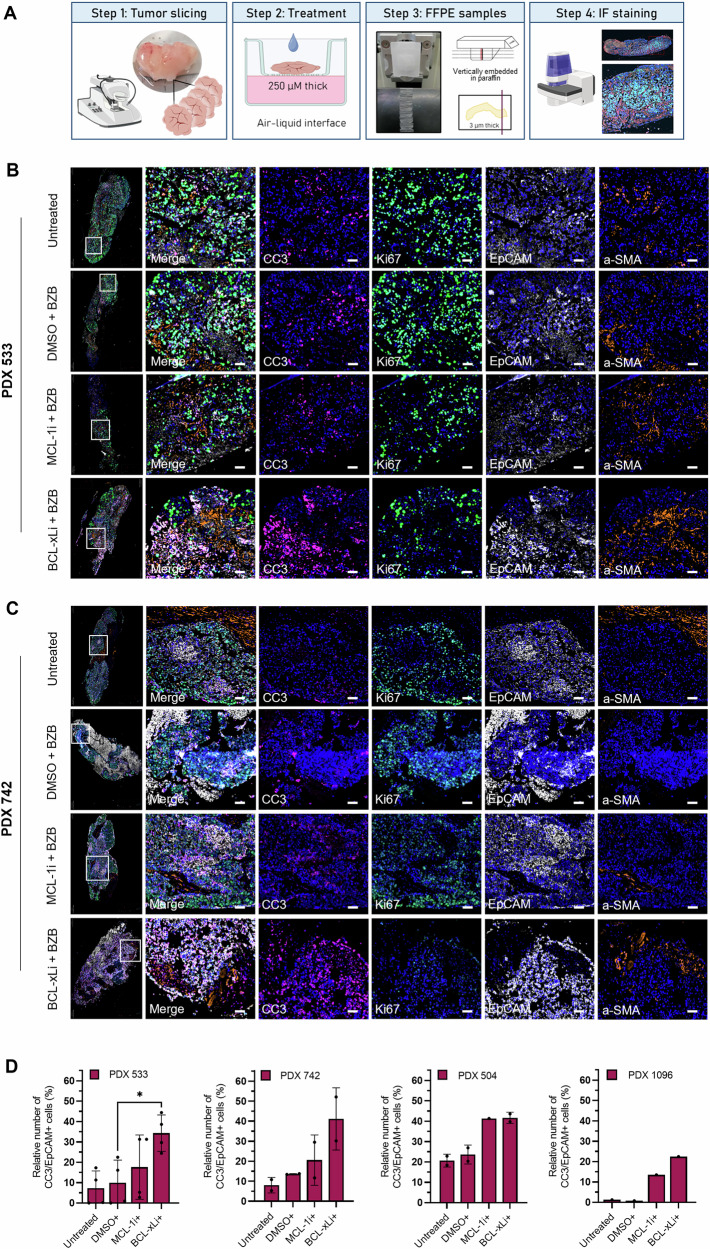
Cetuximab-resistant PDX tumor slices are sensitive towards cell death-inducing agents. **A** Workflow for the generation of tissue slices using a vibratome. Briefly, tumors were cut into 250 µm thick slices and cultivated on top of Millicell© cell culture inserts. Tumor slices were treated with 10 µM BH3-mimetics (MCL-1i or BCL-xLi) in combination with 5 nM bortezomib. In addition, one drop of medium containing the aforementioned drugs was applied on top of each slice. DMSO with bortezomib was used as a control. After 24-48 h, tissue slices were harvested and embedded in paraffin for slicing into 3 µm sections, followed by multiplex immunofluorescence staining. **B, C** Sections of tissue slices derived from the models **B** PDX 533 and **C** PDX 742, and were stained for cleaved caspase 3 (CC3; pink), Ki67 (green), EpCAM (white), a-SMA (orange), and DAPI (blue) after 24 or 48 h of treatment. Scale bars of selected tissue areas (white box) represent 50 μm. **D** Quantification of multiplex immunofluorescence analysis using QuPath. Drug efficacy was determined by calculating the ratio of EpCAM and CC3 double-positive tumor cells to the total number of EpCAM-positive tumor cells. Tissue slices from PDX models 504 (derived from one tumor) and 533 (derived from two tumors) were treated for 48 h, tissue slices from PDX model 742 and 1096 (each derived from one tumor) were treated for 24 h. Data shown as mean ± SD if 2 tissue slices per condition were tested, otherwise one tissue slice per condition was analyzed. Data shown as mean ± SD. Statistical significance was determined using a two-tailed unpaired t-test with Welch´s correction. *p < 0.05. BZB bortezomib, a-SMA alpha-smooth muscle actin.

Using a vibratome, fresh tumors were sectioned into 250 µm slices and cultured on a Millicell® filter support using a previously established protocol [16]. The slices were treated with BH3-mimetics + bortezomib, using DMSO + bortezomib as vehicle control. Untreated samples were cultured in parallel to monitor the tissue slice viability. Following treatment, the tissue slices were vertically embedded in paraffin, sectioned and analyzed by multiplex immunofluorescence staining, using cleaved caspase 3 (CC3) as a marker for cell death (Fig. 6A). Representative images of the PDX models 533 and 742 display increased CC3 levels in response to BCL-xLi + bortezomib, whereas no increase in CC3-positive cells was observed in response to MCL-1i + bortezomib (Fig. 6B, C; respectively). Inhibition of MCL-1 + bortezomib only induced cell death in the PDX models 504 and 1096, as indicated by CC3-positive staining within the EpCAM-positive tumor cell fraction (Fig. 6D, Fig. S12A, B). In contrast, BCL-xLi + bortezomib effectively induced cell death in 20-40% of the tumor cells in the four PDX models, including the two models with oncogenic BRAF mutations (Fig. 6D). Taken together, these findings demonstrate that BCL-xL is a promising therapeutic target for cetuximab-resistant, KRAS wild-type CRC, as shown by the resistant cell line model and in ex vivo tissue slices derived from four PDX models.

## Discussion

Given the heterogeneity of drug evasion mechanisms, there is an urgent need to identify broadly applicable therapeutic strategies targeting drug-resistant cancer cells. Here, we used 2D and 3D cell culture models, along with ex vivo cultured PDX-derived tissue slices to identify and validate therapeutic vulnerabilities of cetuximab-refractory CRC.

The cetuximab-resistant cell lines generated in this study were characterized by persistent MAPK activation, which can be partially explained by HRAS mutations as identified by RNA sequencing analysis. In CRC, activating mutations in HRAS are rather uncommon ( < 2%; [7]). It is possible that the parental cells harbored mutant subclones in a low frequency that expanded under drug pressure, or emerged due to the microsatellite instability in this cell line [2, 8]. Irrespective of the specific activating mutation, MEK inhibitors suppressed the proliferation of CRC cell lines carrying KRAS, BRAF or NRAS mutations, and even induced cell death in HRAS mutant bladder, endometrium, and lung cancer cells [9, 19]. However, blocking MEK1/2 ± cetuximab only slightly inhibited the proliferation of LIM1215-R1/R2 cells, underscoring the involvement of other MEK-independent pathways driving survival, which can be explained by the polyclonal nature of the cell lines. CRC is known for its high intratumoral heterogeneity and the co-existence of molecularly distinct subclones with varying degrees of drug sensitivity [23], which supports the translational importance of a heterogeneous model and the need for therapies targeting common vulnerabilities of CRC.

Due to the potential of BH3-mimetics to induce cell death downstream of oncogenic signaling, they offer a promising therapeutic approach for the treatment of cetuximab-resistant cells. Unlike non-malignant cells, CRC cells were shown to depend mainly on MCL-1 and BCL-xL, while BCL-2 plays a minor role [14]. This also holds true for LIM1215-R1/R2 cells, in which the MCL-1 and BCL-xL proteins were readily detected. However, in line with other reports, we did not observe a strong correlation between BH3-mimetic sensitivity and BCL-xL or MCL-1 expression, suggesting that sensitivity likely depends on multiple factors within the apoptotic pathway [14].

While KRAS mutations can confer resistance to MCL-1 inhibitors by upregulating BCL-xL through the ERK pathway [20], this was not the case in the HRAS-mutant cell model generated here. In fact, sensitivity to MCL-1i monotherapy was elevated compared to LIM1215-parental cells, indicating that oncogenic HRAS does not predict resistance to cell death induction. This assumption is further supported by the pre-treatment experiments with AZD6244 + cetuximab revealing suppression of oncogenic HRAS expression along with reduced sensitivity to BCL-xLi ± bortezomib. This might be explained by a MEKi- induced enrichment of cells with lower BCL-xL dependence, or an adaptive resistance stemming from altered expression of apoptosis regulators. By contrast, the cell death response to MCL-1i + bortezomib remained unchanged in the pre-treated LIM1215-R1/R2 cells, but increased in the parental cells. Nevertheless, despite reacting to the MEKi pre-treatment, LIM1215-R1/R2 cells retained apoptosis competence comparable to the parental cells.

The limited clinical application of BH3-mimetics is mainly due to dose-dependent, off-target side effects, such as thrombocytopenia associated with BCL-xL inhibition [10, 24]. Side effects can be minimized by combinatorial treatments with other agents for dose reduction strategies while preserving therapeutic efficacy Previous studies demonstrated enhanced apoptosis of CRC cells towards BH3-mimetics with proteasome inhibitors, resulting from the stabilization of the pro-apoptotic protein NOXA [20, 21]. Indeed, upon co-treatment with bortezomib, increased cell death was observed in the cetuximab-resistant cells, especially upon MCL-1 inhibition, but BH3-mimetic combination did not achieve additive or synergistic effects at lower doses. Interestingly, while the cells were sensitive to MCL-1i monotherapy in 2D culture, the drug failed to induce cell death in 3D, underscoring the importance of evaluating cell survival in physiologically relevant environments.

Importantly, these promising results were validated in ex vivo cultures of cetuximab-resistant, KRAS wild-type PDX-derived CRC tissues. Tumor slice cultures are physiologically relevant models that preserve the tumor architecture and reflect patient heterogeneity [16]. Although the PDX tissues were not pre-exposed to cetuximab, the molecular alterations conferring resistance are largely preserved, irrespective of the evolving path. Inhibition of BCL-xL + bortezomib potently induced cell death in all models. Cell death was also observed in the PDX models harboring oncogenic BRAF mutations, further supporting that MAPK activation does not prevent responsiveness to BCL-xL inhibition. By contrast, MCL-1i plus bortezomib induced only partial responses in two out of four PDX models, indicating broader applicability for BCL-xL inhibitors.

In conclusion, our findings indicate that cetuximab-resistant CRC cells remain apoptosis-competent and can be effectively targeted by BCL-xLi. While we used co-treatment with bortezomib as a proof-of-principle approach, future studies should explore alternative therapeutic combinations and refine dosing strategies to minimize off-target effects.

## Material and methods

### Antibodies

For flow cytometry, phycoerythrin (PE)-conjugated anti-human Fc antibody was purchased from Dianova (goat IgG anti-Human IgG (Fc)-RPE, 109-115-098; Hamburg, Germany [RRID:AB_2337675]), and rituximab was provided by Roche Diagnostics (Basel, Switzerland). For the generation of resistant cells and the cell characterization, cetuximab was provided by the Robert-Bosch-Hospital Stuttgart, Germany. For immunoblot analysis, antibodies were purchased from Cell Signaling (phospho-EGF Receptor (Tyr1068) (D7A5) XP® rabbit mAb #3777 [RRID:AB_2096270]; EGF Receptor rabbit pAb #2232 [RRID:AB_331707]; p44/42 MAPK (Erk1/2) (3A7) mouse mAb #9107 [RRID:AB_10695739]; phospho-p44/42 MAPK (Erk1/2) (Thr202/Tyr204) rabbit pAb #9101 [RRID:AB_331646]; Cofilin (D3F9) XP® rabbit mAb #5175 [RRID:AB_10622000]; PARP rabbit pAb #9542 [RRID:AB_2160739], Cell Signaling Technology Europe B.V; Frankfurt am Main, Germany), from Dianova (goat IgG anti-mouse IgG (H + L)-HRPO, MinX Hu,Bo,Ho pAb #115-035-062 [RRID:AB_2338504]; goat IgG anti-rabbit (H + L)-HRPO, MinX Hu,Ms,Rt pAb #111-035-114 [RRID:AB_2307391], Hamburg, Germany) and from Sigma-Aldrich (alpha tubulin, clone: DM1A mouse mAb #05-829 [RRID:AB_310035], Taufkirchen, Germany). For multiplex immunofluorescence staining, antibodies were purchased from Agilent (Ki67, clone: MIB-1, mouse mAb #M7240 [RRID:AB_2142367]; EpCAM, clone: BER-EP4 mouse mAb #M0804 [RRID:AB_2335685], Agilent Technologies, Santa Clara, USA), from Cell Signaling (cleaved caspase 3 Asp175 pAb, #9661 [RRID:AB_2341188], Cell Signaling Technology Europe B.V; Frankfurt am Main, Germany) and from Abcam (alpha smooth muscle actin rabbit pAb #ab5694 [RRID:AB_2223021], Cambridge, UK).

### Cell culture and generation of cetuximab-resistant cells

LIM1215-parental and cetuximab-resistant cells (LIM1215-R1/R2) were cultured in growth medium containing RPMI-1640 (Thermo Fisher Scientific, MA, USA) supplemented with 10% fetal bovine serum (FBS, F7524, Sigma-Aldrich) and 1x penicillin/streptomycin (Thermo Fisher Scientific) in a humidified chamber with 5% CO_2_ at 37°C. To generate resistant cell lines, LIM1215 cells were treated for six months with gradually increasing concentrations of clinical-grade cetuximab in growth medium, starting from 0.6 µM to a final concentration of 1.4 µM. The procedure was performed simultaneously in duplicate, resulting in two independent resistant cell lines (referred to as LIM1215-R1 and -R2). After long-term treatment, cells were maintained in culture with 100 nM cetuximab for up to 8-10 passages unless described otherwise. Prior to drug treatments, cetuximab was withdrawn from the culture medium for 2-3 passages to eliminate residual drug effects. LIM1215 cells were purchased from ECACC in 2018 (RRID:CVCL_2574), tested negative for mycoplasma (Lonza, Basel, Switzerland) and the genetic identity was confirmed by SNP profiling (Multiplexion GmbH, Heidelberg, Germany) in 2020.

### Cell viability assay

#### 2D-culture assay

LIM1215 cells were seeded at a density of 1 000 cells per well in 96-well plates. After 24 h, the cells were treated with clinical-grade cetuximab or AZD6244 (S1008, Selleckchem, TX, USA), using PBS or DMSO as respective control. For cellular ATP measurements, the medium was replaced by 50 µl detection reagent (1:1 dilution of growth medium with CellTiter-Glo® 2.0 (Promega, Wisconsin, USA)). The luminescence was determined using a Spark microplate reader (Tecan, Maennedorf, Switzerland).

#### 3D-culture assay

For 3D cultures, 96-well plates were pre-coated with a 1:1 mixture of Matrigel (356231; Sigma-Aldrich) and collagen (PureCol®-S, 5015; Advanced BioMatrix, CA, USA) and incubated for 15 min in a humidified incubator (37°C, 5% CO₂). A total of 2 000 cells/well were seeded in growth medium supplemented with 2% Matrigel. After 48 h, cells were treated with BH3-mimetics targeting MCL-1 (SML1932; Sigma-Aldrich) or BCL-xL (SML3162; Sigma-Aldrich), either alone or in combination with 5 nM bortezomib (F1200, TX, UBPBio), using DMSO as a control. After 48 h of treatment, CellTiter-Glo® 3D reagent (Promega) was added to each well in a 1:1 ratio with the growth medium. Luminescence was measured using a Spark microplate reader (Tecan). 3D culture growth was assessed using the Incucyte live-cell imaging system (Sartorius, Göttingen, Germany) and spheroids were imaged every 2 h. Spheroid growth was determined after drug exposure using the Incucyte analysis tool “Brightfield Object Total Area”, followed by normalization to the time point 0 h.

### Cell death assay

#### 2D-culture assay

Cell death was determined by co-staining of cells with Annexin V-GFP (produced in-house) and propidium iodide (PI; Sigma-Aldrich). Parental and resistant LIM1215 cells (15 000 cells/well) were seeded in 96-well plates and treated with BH3-mimetic drugs either alone or in combination with 5 nM bortezomib. After 24 h of treatment, attached cells were harvested by trypsinization and collected with the supernatant for cell death analysis. Cells were stained with 10 µg/mL PI and Annexin V-GFP in binding buffer (140 mmol/L NaCl, 2.5 mmol/L CaCl_2_, 10 mmol/L HEPES, pH 7.4) for 10 min at RT, followed by flow cytometry using the MACSQuant® Analyzer (Miltenyi Biotec, Bergisch Gladbach, Germany). The proportion of dead cells was determined by the combination of positive staining for Annexin V-GFP and double-positive staining for Annexin V-GFP/PI.

#### 3D-culture assay

Spheroids were generated as mentioned before and treated for 48 h with BH3-mimetics +/- 5 nM bortezomib, supplemented with 0.5 µg/mL PI. Cell death was assessed by PI signal intensity over time using the Incucyte live-cell imaging system (Sartorius, Göttingen, Germany) and spheroids were imaged every 2 h. Cell death was determined by quantification of the PI signal intensity after drug exposure using the Incucyte analysis tool “All Brightfield Relative Total Red Integrated Intensity”, followed by normalization of the PI signal to day 0. For cell death analysis by flow cytometry, 3D cultures were harvested by trypsinization and collected with the supernatant for cell death analysis. Cells were stained with Annexin V/PI and cell death was determined by flow cytometry using the MACSQuant® Analyzer (Miltenyi Biotec, Bergisch Gladbach, Germany).

### Antibody cell surface binding

Parental and resistant LIM1215 cells were incubated with cetuximab for 1 h at 4°C, using rituximab as a negative binding control. Antibodies were diluted in PBS containing 2% FBS and 0.02% sodium azide and detected with an R-PE-labeled anti-human Fc antibody. Antibody binding was investigated by flow cytometry (MACSQuant® Analyzer, Miltenyi Biotec), followed by analysis using FlowJo Version 10 (Tree Star). Relative MFI was calculated as follows: relative MFI = ((MFI_sample_ - (MFI_detection_-MFI_cells_))/MFI_cells_).

### Immunoblotting

Cells were lysed with RIPA buffer (25 mmol/L Tris pH 7.4, 1% Nonident P-40, 0.5% sodium deoxycholate, 150 mmol/L NaCl, 0.1% SDS, PhosSTOP (Merck, Darmstadt, Germany), cOmplete protease inhibitors (Merck)). Protein concentrations were determined using the Bio-Rad DC protein assay (CA, USA). Lysates were loaded on 4–12% NuPAGE® Novex Bis-Tris gels (Thermo Fisher Scientific) and transferred to membranes using iBlot® Gel Transfer Stacks (Thermo Fisher Scientific). After membrane blocking (0.5% blocking reagent (Roche Diagnostics) and 0.05% Tween-20 in PBS), membranes were incubated with primary and HRP-labeled secondary antibodies in blocking solution. Mild membrane stripping (0.2 M glycine, 1% SDS, 1% Tween, pH 2.2) was performed at room temperature for 20 min, followed by reprobing with antibodies against total ERK/EGFR (Fig. 1E). For signal detection, membranes were incubated with SuperSignal chemiluminescent substrates (Thermo Fisher Scientific) and visualized with the FUSION SL system (Vilber Lourmat, Collégien, France). Signals were determined using the software FusionCapt Advance (Vilber Lourmat) and target protein levels were normalized to the loading control. Uncropped western blot images are displayed in the section “Supplemental Material”.

### RNA-sequencing library preparation and sequencing analysis

Total RNA of LIM1215 cell lines was isolated using the miRNeasy Mini Kit (217004, Qiagen) according to the manufacturer’s instructions. RNA integrity was assessed by gel electrophoresis, and libraries were prepared using 1 µg of RNA with the Illumina Stranded mRNA Prep Ligation Kit (1000000124518 v03, Illumina, CA, USA), following the manufacturer’s protocol. After library preparation, library concentration was measured using a Qubit Fluorometer (Invitrogen, Thermo Fisher Scientific) with the Qubit 1X dsDNA High Sensitivity Kit (Q33231, Thermo Fisher Scientific). Library size was assessed using TapeStation (Agilent) with the High Sensitivity D1000 Sample Buffer (5190–6504, Agilent) and High Sensitivity D1000 ScreenTape (5067–5584, Agilent). Paired-end sequencing was performed at the Robert Bosch Center for Tumor Diseases (Stuttgart, Germany) using the Illumina NextSeq 2000 system.

Total RNA samples of PDX tumors were digested by DNaseI (NEB, MA, USA) and purified by oligo-dT beads (Dynabeads mRNA purification kit, Invitrogen, Thermo Fisher Scientific). Poly(A)-containing mRNAs were fragmented into 200–250 bp with fragment buffer (Ambion, Thermo Fisher Scientific). Double strand cDNA synthesis and sequencing libraries were prepared and validated following the sequencing provider’s RNA-sequencing protocols. Sequencing was performed at Novogene China using Illumina HiSeq-2000/2500/4000 in paired-end reads.

### RNA-sequencing bioinformatics analysis

For the analysis of LIM1215 cell lines, paired-end sequencing reads were aligned to the human reference genome (hg38) using STAR (v2.7.9a; [25]). Quantification of reverse (sense strand) reads was performed using htseq-count (htseq/0.9.1; [26]). Mutations in sequence data of LIM1215 cell lines were analyzed and visualized using the Integrated Genome Viewer (version 2.15.2, GRCh38/hg38) For the bioinformatic analysis of the PDX models, xenoma (v1.00), with a xenome-index built from GRCh38 (v90) and GRCm38 (v90), was used to classify paired-end reads from xenograft samples, and mouse-derived RNA was identified and filtered out to obtain human-derived reads. Only human reads were considered for further analysis and paired-end reads were mapped to the human reference genome (GRCh38, v90) using HISAT2 (v2.1.0; [27]). Mapped reads were matched to GENCODE (v27) and processed using StringTie (v1.3.4; [28]) to obtain raw counts and normalized gene expression values (counts, FPKM and TPM values).

Differential gene expression analysis of either the resistant LIM1215 cell lines or the PDX models was carried out with DESeq2 (v1.38.3; [29]), using the LIM1215-parental cell line as a reference for both model systems. Genes with low expression ( < 10 counts) were filtered out and the differentially expressed genes (DEGs) were identified using the Wald test. To account for potential variance and biases between samples, the resulting DEGs were further processed using the lfcShrink function in DESeq2. Multiple testing correction was performed using the Benjamini-Hochberg method to control the false discovery rate (FDR). DEGs with a log2(fold change) > 1.5 and an adjusted *p*-value < 0.01 were considered for further analysis and gene annotation clustering. Up- and down-regulated genes shared by cetuximab-resistant LIM1215 lines and PDX models were selected to determine top annotation clusters using the DAVID Functional Annotation Clustering Tool [30]. Principal component analysis computations and plotting were performed with the PCA function from the R package FactoMineR (v2.11; [31]). The volcano plots were generated with the R package EnhancedVolcano. Only the genes with the smallest adj. p-values and highest log2FCs are labeled [32].

### Xenograft models

The tumor xenografts derived from surgical specimens obtained from cancer patients. Following excision at surgery, tumor pieces were implanted subcutaneously into 4–8 week-old female immunodeficient mice (NSG or NMRI nu/nu) and are therefore referred to as patient-derived tumor xenografts (PDXs). Following their primary implantation into immunodeficient mice (passage 1), the tumor xenografts were passaged until establishment of a stable growth pattern and master stocks were generated. Tumor fragments obtained from xenografts in serial passage in immunodeficient mice were used to implant recipient animals with bi- or unilateral tumor implants subcutaneously in the flank(s). The absolute tumor volumes were determined by two-dimensional measurement with a digital caliper (S_Cal EVO Bluetooth, Switzerland) on the day of randomization and then twice weekly. Tumor volumes were calculated according to the formula: Tumor volume = (l × w²) × 0.5 where l = length and w = width (in mm) of the tumor.

For treatment experiments, the animals were monitored until the tumor implants reached the volume criteria of 50–200 mm^3^, preferably 80–150 mm^3^ in a sufficient number of animals. Mice were assigned to groups, aiming a comparable group median and mean tumor volumes (randomization without blinding). The day of randomization was designated as day 0 of the experiment. Treatment experiments were terminated when the tumors reached a size of ≥2000mm^3^ at day of measurement. For the generation of tissue slice cultures, the animals were sacrificed when the tumor volume reached a maximal size of 700–800 cm^3^, followed by the extraction of the PDX models further processing.

### Generation and treatment of tumor tissue slices

PDX models (CXF 504, 533, 742 and 1096) were obtained from Charles River Laboratories (Freiburg, Germany). Whole exome sequencing data for all PDX models are publicly available through the Charles River cancer model portfolio (Patient-Derived Xenograft: PDX Models | Charles River). Tumors were shipped overnight in ice-cold MACS tissue storage solution (Miltenyi Biotec B.V. & Co. KG, Gladbach, Germany) and immediately prepared for tissue slicing upon arrival. Tumors were embedded in 3D-printed molds and supported with 4% agarose in PBS for slicing. The agarose blocks were mounted on a specimen disk using cyanoacrylate adhesive. Using the vibratome VT1200S, 250 µm thick tissue slices were generated (Leica Biosystems, Wetzlar, Germany). Necrotic slices were excluded for further screenings. For cultivation, the slices were placed on Millicell® Cell Culture inserts (Merck, PTFE, pore size 0.4 μm) in 6-well plates. The slices were incubated in a humidified chamber with 5% CO_2_ at 37°C in RPMI-1640 (Thermo Fisher Scientific) supplemented with 10% fetal bovine serum (FBS, Sigma-Aldrich), penicillin (100 U/ml; Gibco, Thermo Fisher Scientific), streptomycin (100 μg/ml; Gibco, Thermo Fisher Scientific) and glutamine (2 mM; Gibco, Thermo Fisher Scientific). For the drug treatments, up to 2 slices from different tumor positions were selected for each drug combination. The medium was replaced daily, containing 10 µM of BH3-mimetic drugs targeting MCL-1 (Sigma-Aldrich) or BCL-xL (Sigma-Aldrich) in the presence of 5 nM bortezomib, with DMSO as control. Additionally, one drop of medium containing the drugs or control was applied on top of each slice. Slices were harvested after 24–48 h and fixed in 10% formaldehyde (Carl Roth, Karlsruhe, Germany). The slices were further processed at the Robert-Bosch-Hospital (Stuttgart, Germany) using standardized operating procedures for paraffin blocks, followed by vertical embedding in paraffin.

### Multiplex immunofluorescence microscopy and image analysis

Paraffin-embedded slices were cut in 3 µm thick sections using a microtome (Leica). Samples were mounted on Superfrost Plus microscope glass slides (Thermo Fisher Scientific), with control and treated slices from each tumor placed and processed on the same slide to ensure uniform staining conditions. The slides were then incubated at 56°C for 3 h. Tissue processing and staining were conducted using an optimized protocol for the Opal 6-Plex Manual Detection Kit (NEL861001KT, Akoya Biosciences, MA, USA). Briefly, tissue sections were deparaffinized using NeoClear (Merck) and rehydrated through a descending alcohol series. Slides were subsequently fixed in 4% ROTI®Histofix (Carl Roth), followed by antigen retrieval in a pH 6.0 buffer for all antibodies. Antibody incubation steps were carried out at room temperature. After blocking, the samples were incubated with the primary antibody for 30 min, followed by a 10-min incubation with the secondary antibody (Opal Poly HRP ms+rb). Opal fluorophores (1:100) were applied for 10 min. These steps were sequentially repeated for each antibody used. Tissue slices were stained for EpCAM (1:100), alpha-SMA (1:200), Ki67 (1:75), cleaved caspase 3 (1:50) and counterstained with DAPI. Imaging of the slices was conducted using the PhenoImager™ Fusion (Akoya Biosciences).

The imaging analysis software QuPath was used to determine the cell death response of the cancer cells [33]. The quantification was conducted in whole tissue sections. Within the sections, the DAPI staining was used to determine the total number of cells. All images were manually reviewed to ensure the exclusion of incorrectly identified objects. The mean percentage of cleaved caspase 3-positively stained tumor cells (dead cells) was calculated relative to the total number of EpCAM-positive tumor cells to determine the drug efficacy across all tumor slices.

### Statistical data analysis

Data are presented as the mean ± SD as indicated in the respective figure legend. ‘n’ refers to the number of independent experiments. Statistical analysis was performed using Microsoft Excel 2025 (Microsoft Corporation, Redmond, WA, USA) and GraphPad Prism 8.0 (GraphPad Software, Inc., San Diego, CA, USA). The corresponding tests and number of replicates in each experiment are indicated in the figure legends. A *p*-value below 0.05 was considered statistically significant, statistical significance is indicated as follows: not significant (ns) for *p* > 0.05, * for *p* < 0.05, ** for *p* < 0.01, *** for *p* < 0.001, and **** for *p* < 0.0001.

## Supplementary information

Supplemental Figures

Supplemental Tables

Supplemental material and methods

Uncropped western blots

## Data Availability

The RNA-sequencing data for LIM1215-parental and -R1/R2 cells are available on the GEO website under accession number GSE299943. The RNA-sequencing data for the PDX models are available from Charles River Laboratories (Freiburg, Germany) upon request. Furthermore, uncropped western blots performed in this study are included in this publication. All data and materials reported in this publication will be shared by the lead contact upon request.

## Code availability

The codes for the RNA sequencing analysis will be shared by the lead contact upon request.
