# Supplementary material for: BCL-xL as a therapeutic target in cetuximab-refractory colorectal cancer
Source: Cell Death Dis. 2026 Jan 31;17(1):187. doi: 10.1038/s41419-026-08434-5 (PMC12876907; doi:10.1038/s41419-026-08434-5)
Supplement: Supplementary file 1 — Supplemental Figures [file 41419_2026_8434_MOESM1_ESM.pdf]

Figure S1.

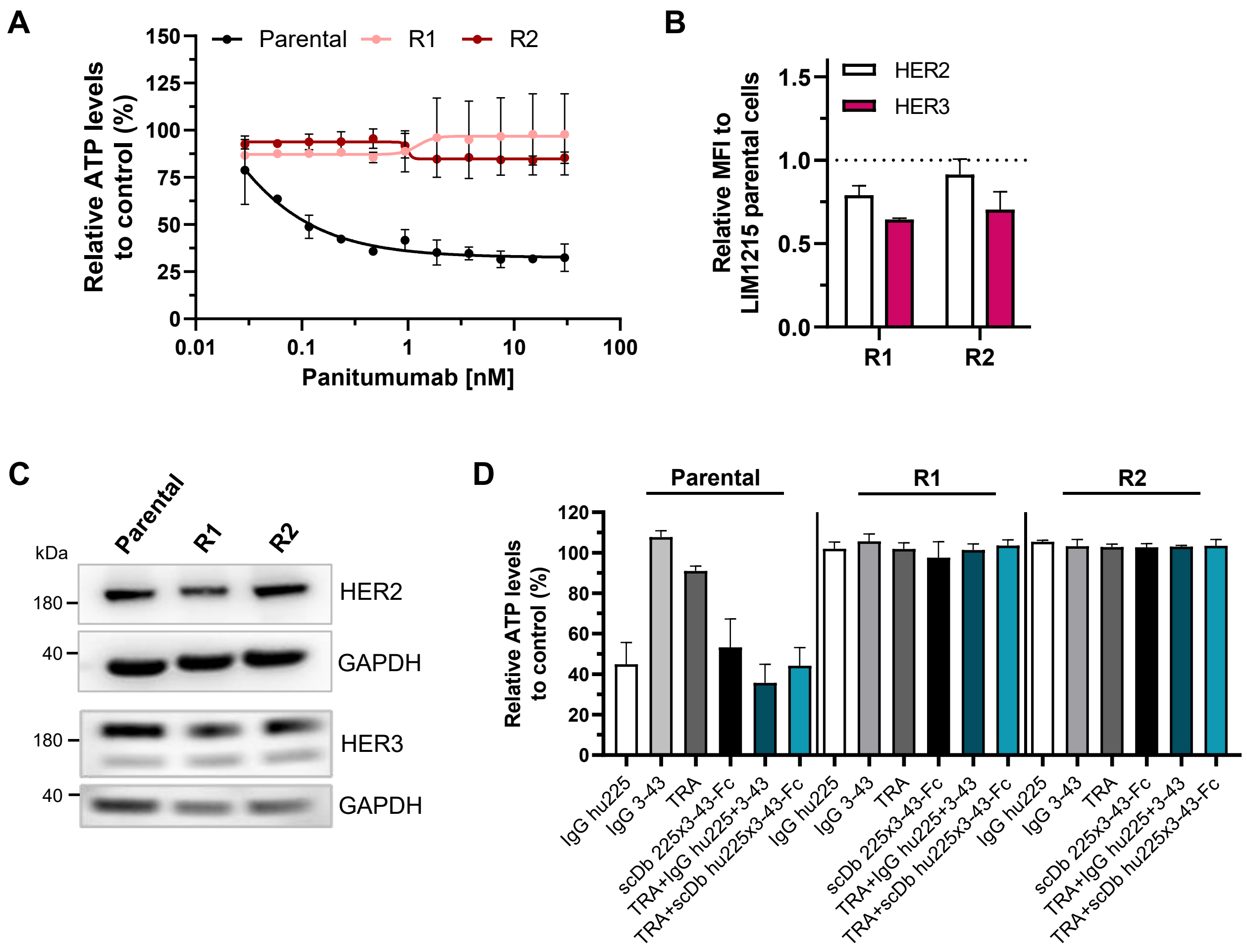

Figure S1. LIM1215-R1/R2 cells are resistant to inhibition of ErbB/HER family members. (A) ATP levels of LIM1215 cell lines after 4 days of treatment with increasing panitumumab concentrations, shown relative to PBS control. Values represent the mean ± SD of n = 2. (B) Binding analysis of HER2 and HER3 antibodies to LIM1215 cell lines by flow cytometry. Data shown as mean fluorescence intensity relative to parental LIM1215 cells (mean ± SD; n = 2). (C) Total HER2 and HER3 protein levels in whole-cell extracts of untreated LIM1215 cell lines. GAPDH was included as a loading control. (D) ATP levels of LIM1215 cell lines in response to a 5-day treatment with monospecific antibodies targeting EGFR (IgG hu225), HER2 (TRA), and HER3 (IgG 3-43), the bispecific antibody targeting both EGFR and HER3 (scDb hu225x3-43-Fc) or a combination of the antibodies, relative to PBS control. ATP levels were determined using the CellTiter-Glo® 2.0 assay (mean ± SD; n = 3). MFI, mean fluorescence intensity; TRA, trastuzumab.

Figure S2.

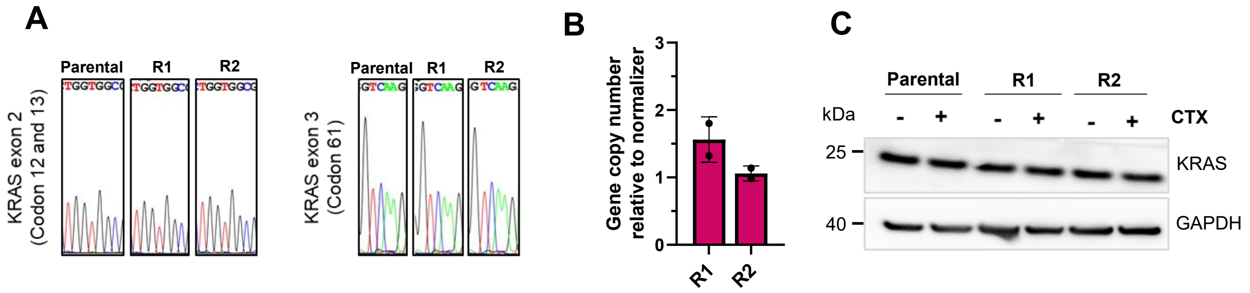

Figure S2. Analysis of KRAS status in cetuximab-resistant LIM1215 cell lines. (A) KRAS exon 2 and exon 3 are wild-type in resistant and parental LIM1215 cells according to Sanger sequencing electropherograms. (B) Gene copy number of resistant cells compared to parental LIM1215 cells. Primers targeting the centromeric region of chromosome 12 were used to normalize the data for aneuploidy. Values represent the mean ± SD of n = 2. (C) KRAS protein levels in cetuximab-resistant and parental LIM1215 cells. Cells were treated with 100 nM cetuximab for 24 hours before extraction of whole-cell lysates for immunoblotting. GAPDH was included as a loading control. CTX, cetuximab.

Figure S3.

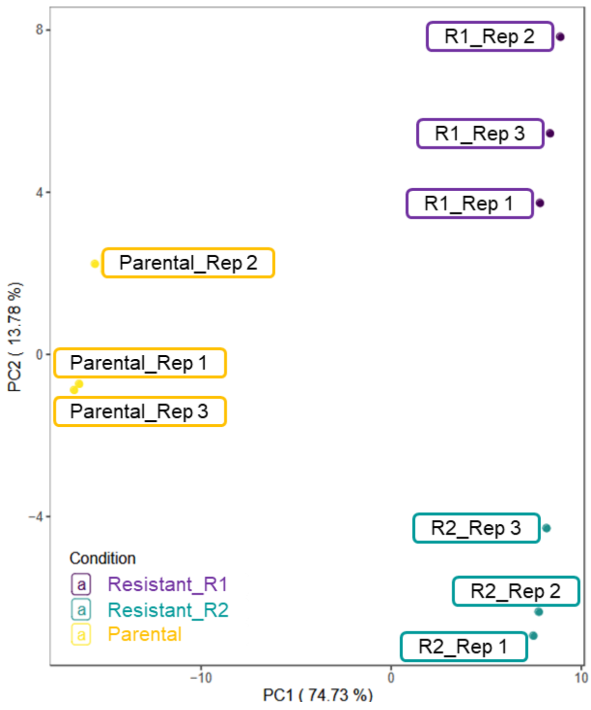

Figure S3. Principal component analysis (PCA) of cetuximab-resistant LIM1215 cells compared to parental cells. Transcriptome analysis of LIM1215-parental and LIM1215-resistant cells was performed by RNA sequencing under basal, untreated culture conditions. Three technical replicates (Rep 1-3) were included per cell line. Gene expression variability of the cetuximab-resistant LIM1215-R1/R2 cells was compared to parental LIM1215 cells. The PCA plot displays the first two principal components (PC1 and PC2), accounting for the majority of the variance in the dataset. Each dot represents a technical replicate, with different colors indicating the respective cell lines.

Figure S4.

A **LIM1215-R1 vs. Parental** **B** **LIM1215-R2 vs. Parental**

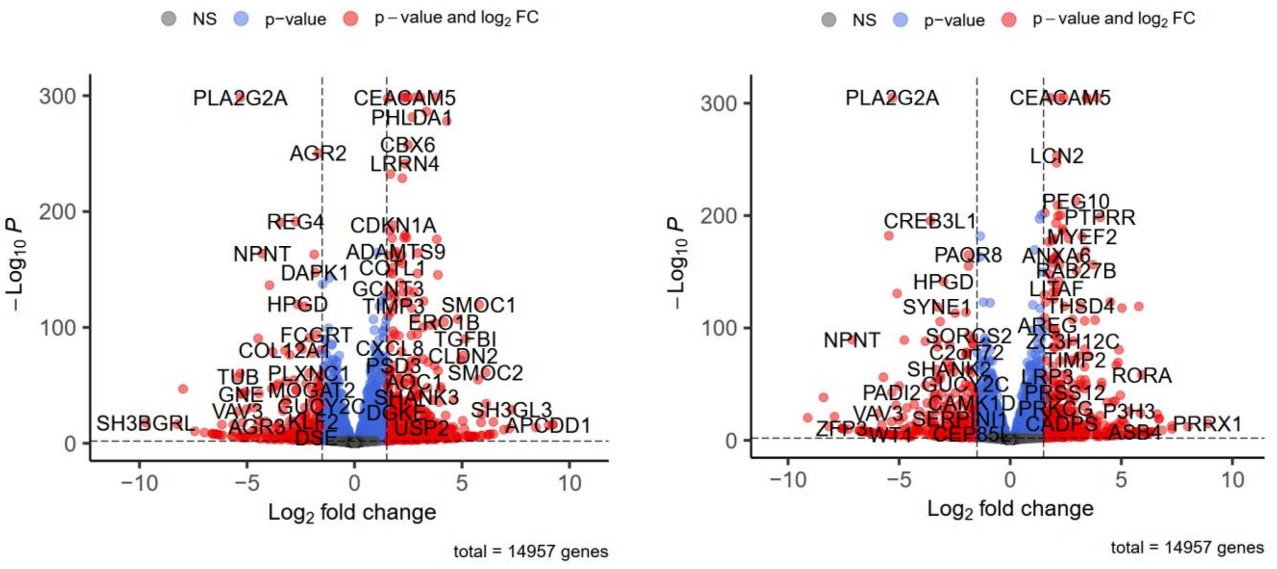

Figure S4. Volcano plot representation of differential gene expression analysis in the LIM1215-parental versus LIM1215-R1 or LIM1215-R2 cell line. Red and blue dots indicate genes with a significantly increased or decreased gene expression, while grey dots indicate no significant changes in gene expression in LIM1215-R1/R2 cells compared to the parental cell line. The y-axis represents the significance of gene expression, and the x-axis represents the log2 fold change.

Figure S5.

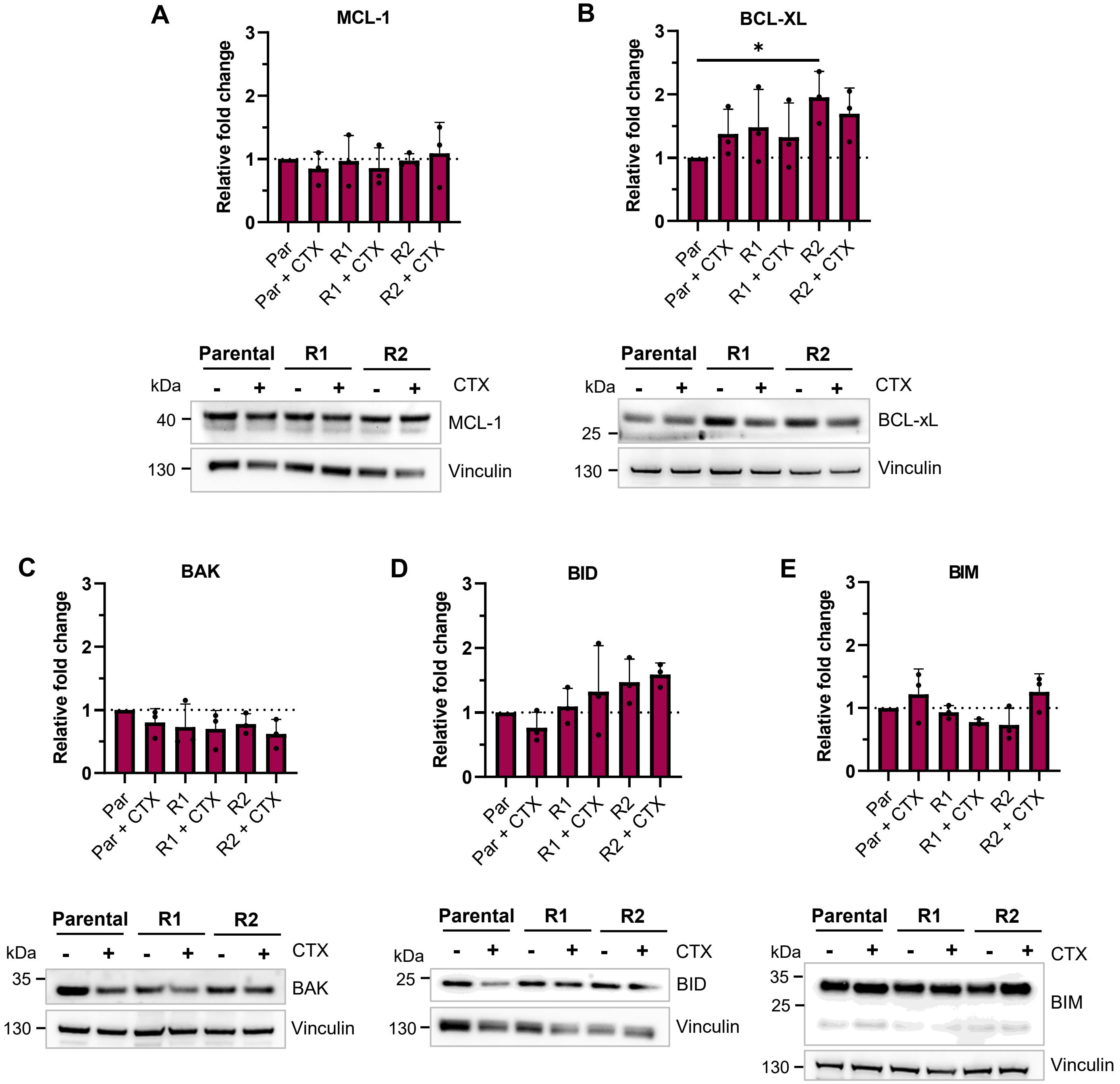

Figure S5. Protein levels of apoptotic regulators under basal culture conditions and cetuximab treatment. (A) MCL-1, (B) BCL-xL, (C) BAK, (D) BID, and (E) BIM protein levels in LIM1215-parental and cetuximab-resistant cells. Protein expression was determined under basal culture conditions in growth medium or in combination with 50 nM cetuximab for 24 hours before extraction of whole-cell lysates for immunoblotting. Vinculin was included as a loading control. Untreated LIM1215-parental cells served as reference to determine the relative fold change. Values represent the mean ± SD of n = 3. Statistical significance was determined using a two-tailed unpaired t-test with Welch´s correction. *$p < 0.05$. Par, parental; CTX, cetuximab.

Figure S6.

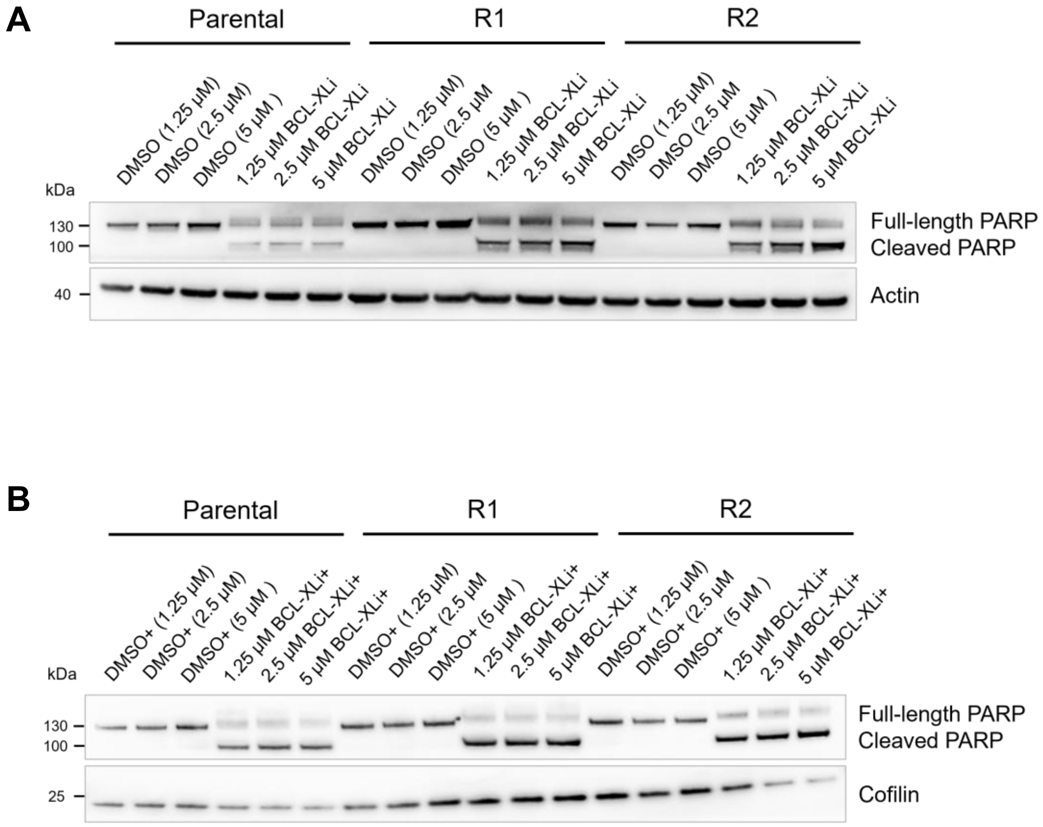

Figure S6. Cell death response of LIM1215-parental and LIM1215-R1/R2 cells upon exposure to increasing concentrations of BCL-xLi ± bortezomib. The LIM1215 cell lines were treated for 24 hours with the BH3-mimetic drug (A) BCL-xLi alone or (B) in combination with 5 nM bortezomib (addition indicated with +). Whole-cell lysates were extracted for immunoblotting to investigate the protein levels of full-length PARP and cleaved PARP. Actin and cofilin were included as loading controls.

Figure S7.

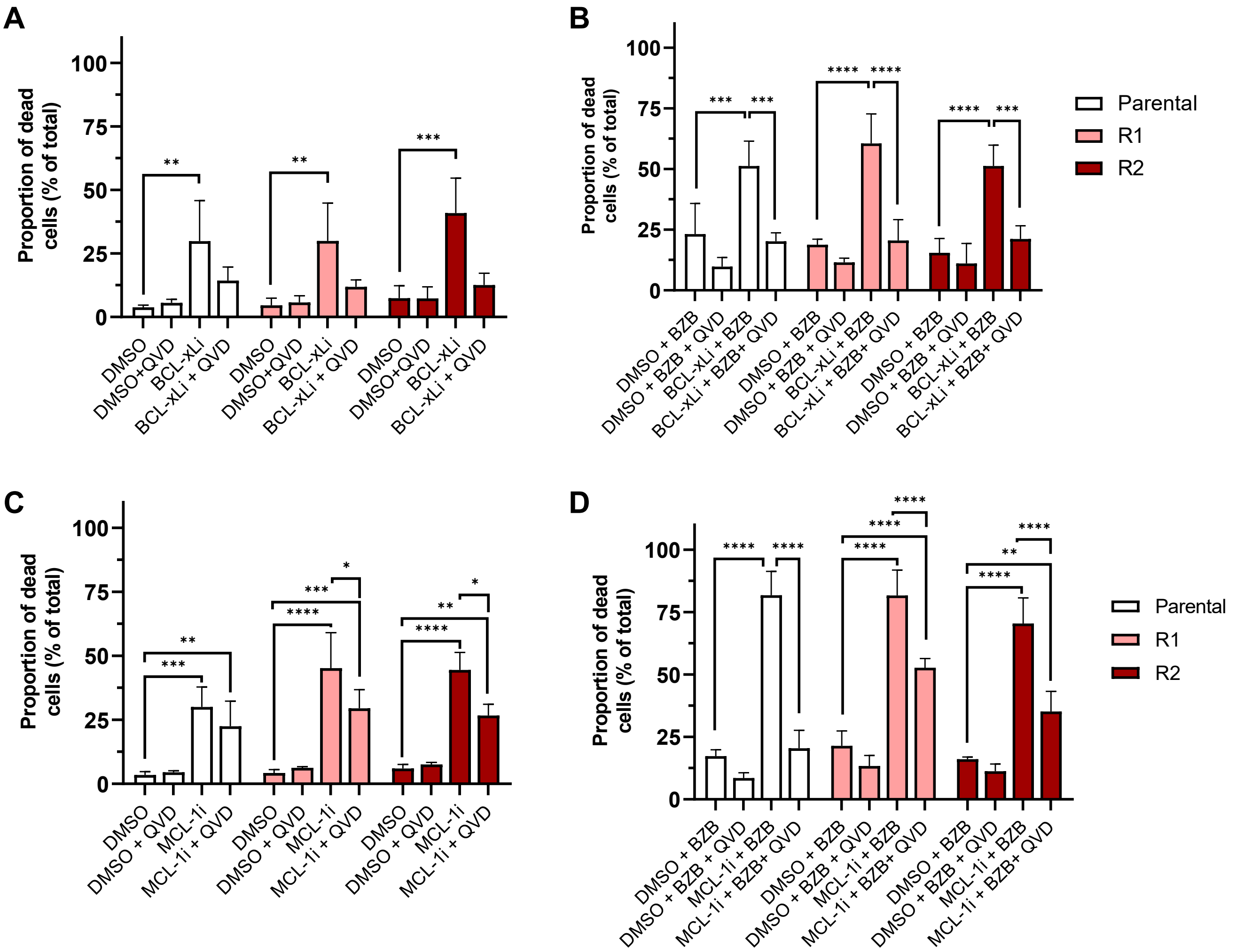

Figure S7. Differential caspase dependence of cell death induced by BCL-xLi and MCL-1i. Cell death analysis of LIM1215 cell lines after treatment with 10 μM BH3-mimetic drugs ± bortezomib in the presence of the pan-caspase inhibitor Q-VD-Oph for 24 hours. Cells were stained with Annexin V-GFP and PI, and the cell death response was analyzed by flow cytometry. The proportion of dead cells was determined by combining the proportion of Annexin V+/PI+ cells and Annexin V+ only cells. (A) Treatment with BCL-xLi ± 10 μM Q-VD-Oph. (B) Treatment with BCL-xLi and 5 nM bortezomib ± 10 μM Q-VD-Oph. (C) Treatment with MCL-1i ± 10 μM Q-VD-Oph. (D) Treatment with MCL-1i and 5 nM bortezomib ± 10 μM Q-VD-Oph. Values represent the mean ± SD of n = 3. Statistics: Two-way ANOVA with Tukey post-hoc test. *$p < 0.05$, **$p < 0.01$, ***$p < 0.001$, ****$p < 0.0001$. QVD, Q-VD-Oph; BZB, bortezomib; MCL-1i, A-1210477; BCL-xLi, A-1155463.

Figure S8.

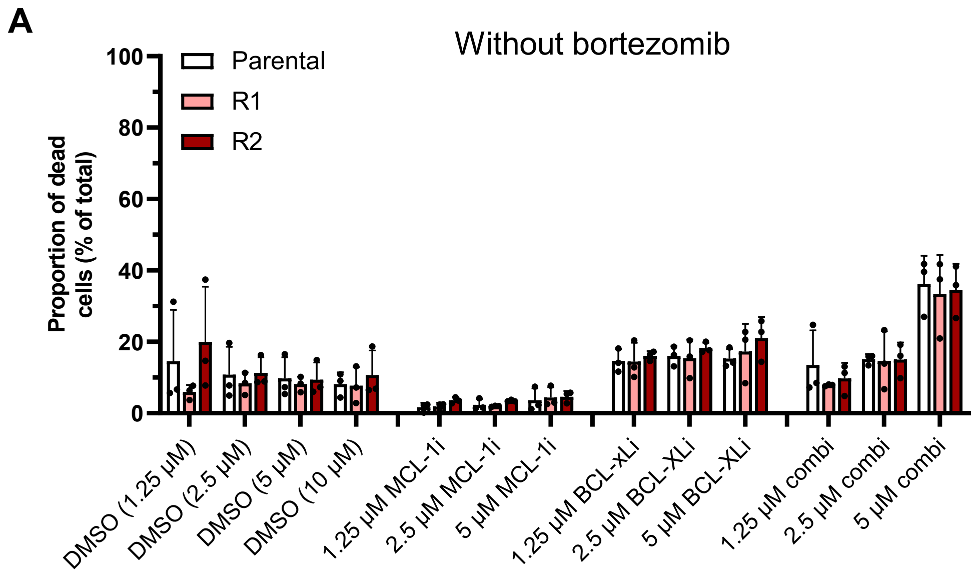

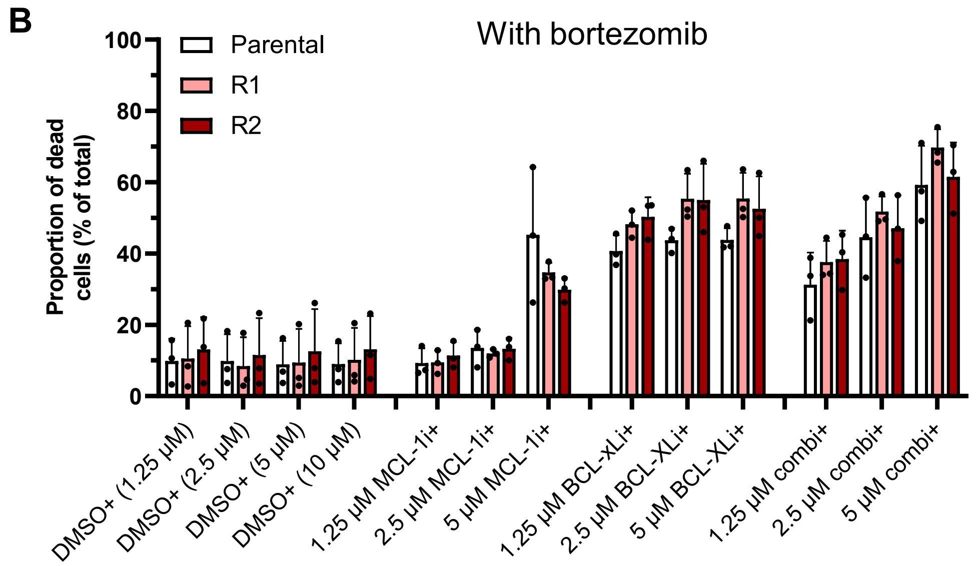

Figure S8. Combinatorial BH3-mimetic drug treatment ± bortezomib of parental and cetuximab-resistant LIM1215 cell lines. Cell death was assessed by flow cytometry after 24 hours of treatment with the BH3-mimetic drugs MCL-1i and BCL-xLi, administered either individually or in combination, with or without 5 nM bortezomib (addition indicated with +). DMSO ± bortezomib served as the vehicle control. Annexin V/PI staining was used to determine the proportion of dead cells (Annexin+/PI+ double-positive and Annexin+ cells). Values represent the mean ± SD of n = 3.

Figure S9.

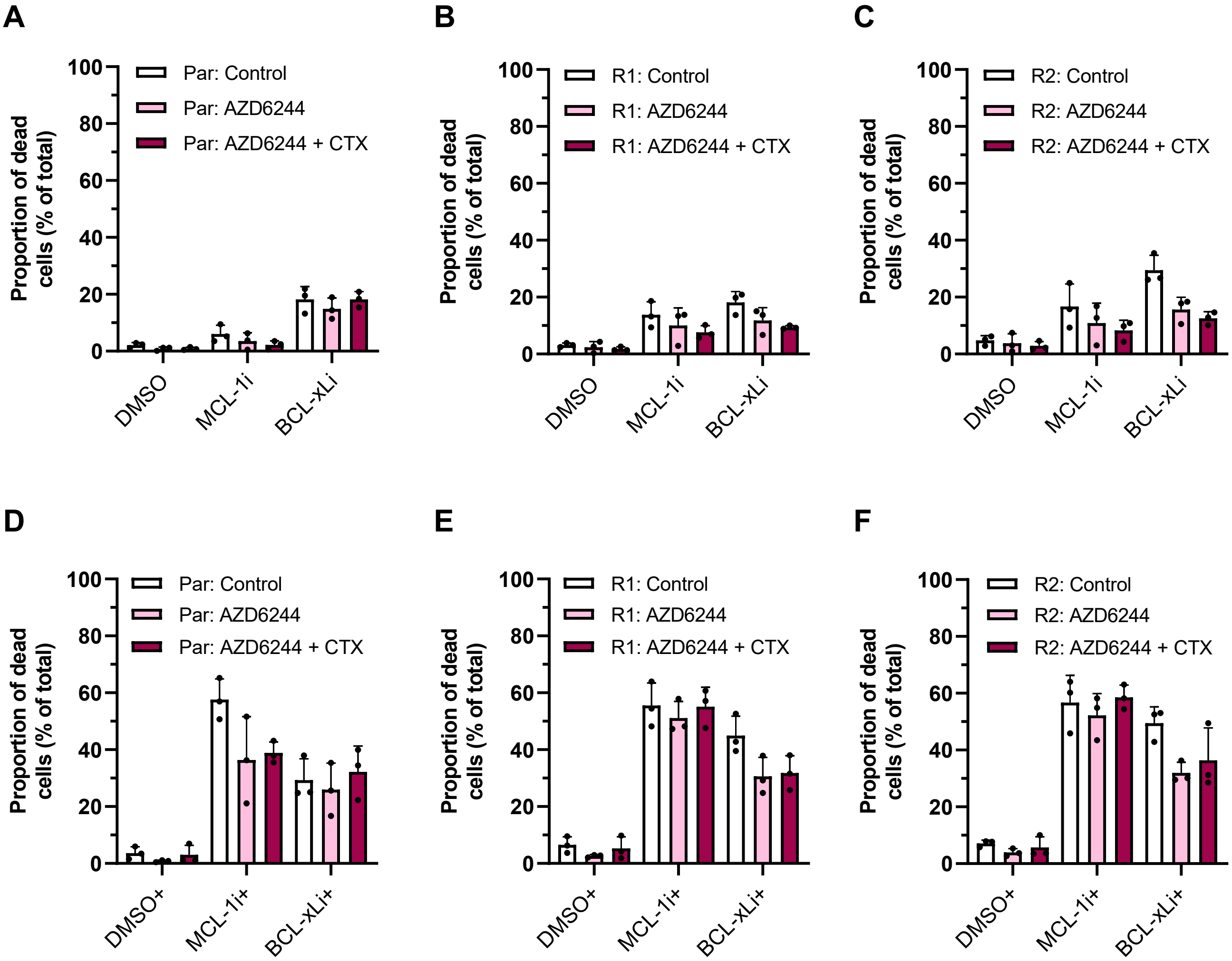

Figure S9. BH3-mimetic sensitivity of LIM1215 cell lines after pre-treatment with AZD6244 ± cetuximab. The cell lines LIM1215-parental and LIM1215-R1/R2 were pre-treated with 250 nM AZD6244 ± 50 nM cetuximab for seven days, with DMSO ± PBS as the control. Afterwards, cell death sensitivity to 10 µM MCL-1i or BCL-xLi was assessed by flow cytometry after 24 hours of treatment. The drugs were applied either as (A-C) monotreatment or (D-F) in combination with 5 nM bortezomib (addition indicated with +). DMSO ± bortezomib served as the vehicle control. Annexin V/PI staining was used to determine the proportion of dead cells (Annexin+/PI+ double-positive and Annexin+ cells). Values represent the mean ± SD of n = 3. Par, parental; CTX, cetuximab.

Figure S10.

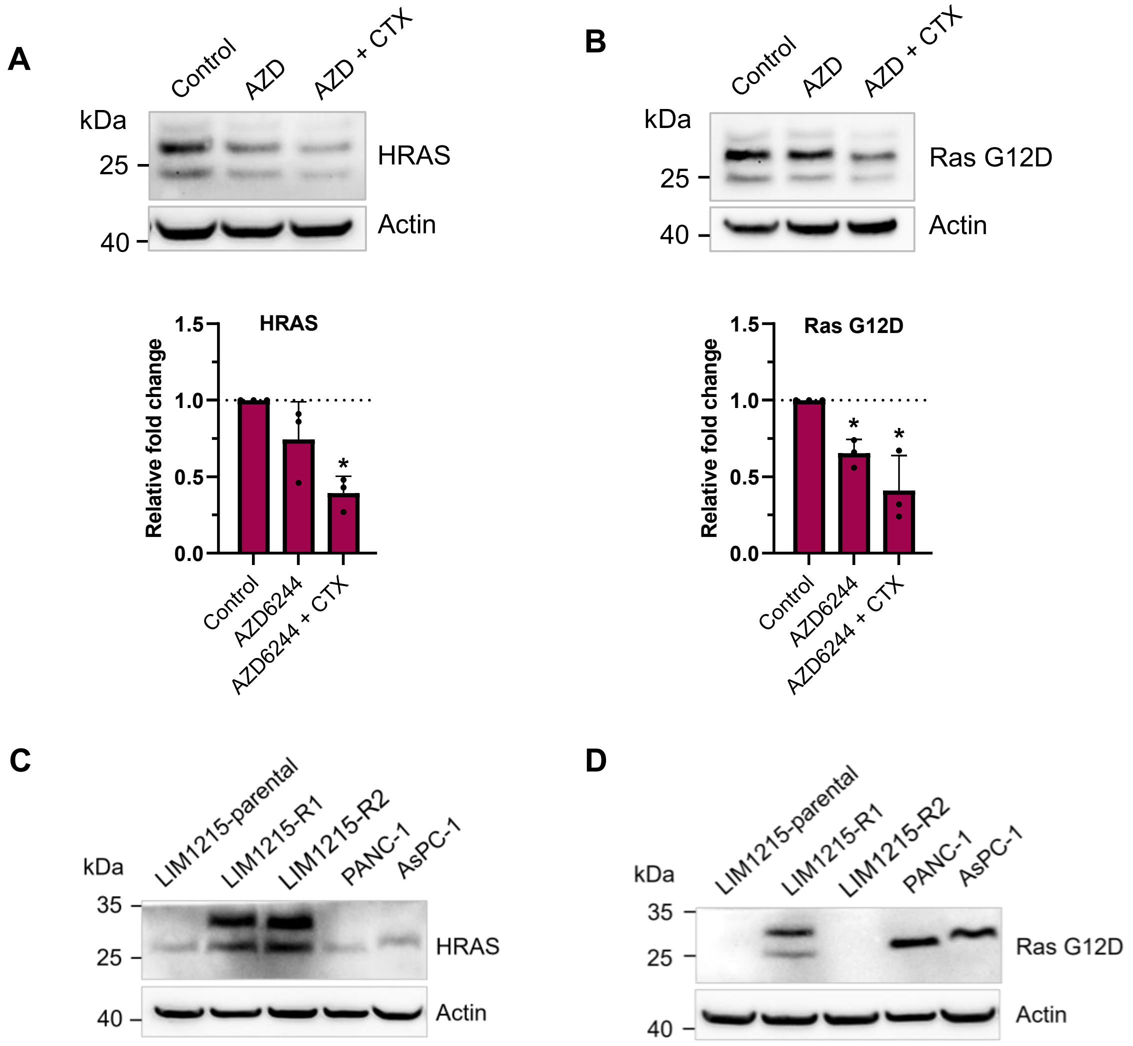

Figure S10. HRAS and Ras G12D protein levels in LIM1215-R1 after seven days of treatment with AZD6244 ± cetuximab. Western Blot analysis of (A) HRAS and (B) Ras G12D protein levels in the cetuximab-resistant cell line LIM1215-R1 under MEK inhibition. Cells were treated with 250 nM AZD6244 (AZD) ± 50 nM cetuximab (CTX) for 24 hours before extraction of whole-cell lysates for immunoblotting. DMSO was used as the vehicle control. Actin was included as a loading control. Treated cells were statistically compared to the control treatment. Western blot analysis of (C) HRAS and (D) Ras G12D protein levels in LIM1215-parental and LIM1215-R1/R2 cells alongside control cell lines under basal culture conditions. Of note, only LIM1215-R1 cells harbored the HRAS G12D mutation, whereas LIM1215-R2 harbored an HRAS Q61H mutation. PANC-1 and AsPC1 cells were included as KRAS G12D positive controls. Whole-cell lysates were extracted for immunoblotting, and actin was included as a loading control. Values represent the mean ± SD of n = 3. Statistical significance was determined using a two-tailed unpaired t-test with Welch´s correction. *$p < 0.05$.

Figure S11.

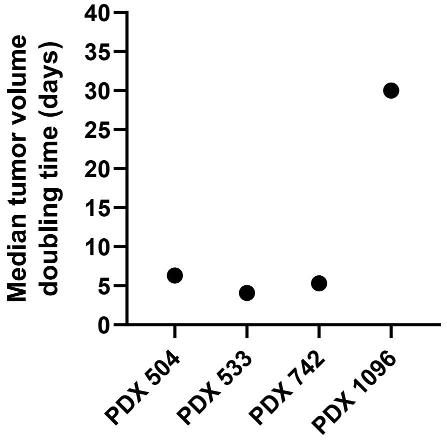

	CTX resistance score (%)	Median tumor volume doubling time (days)
PDX 504	76	6.3
PDX 533	65	4.1
PDX 742	62	5.3
PDX 1096	91	30

Figure S11. Cetuximab-resistant score and *in vivo* tumor volume growth of PDX models. Median tumor volume doubling time of selected cetuximab-resistant, KRAS wild-type PDX models 504, 533, 742, and 1096. The cetuximab resistance score was determined relative to treatment with control vehicle. CTX, cetuximab.

Figure S12.

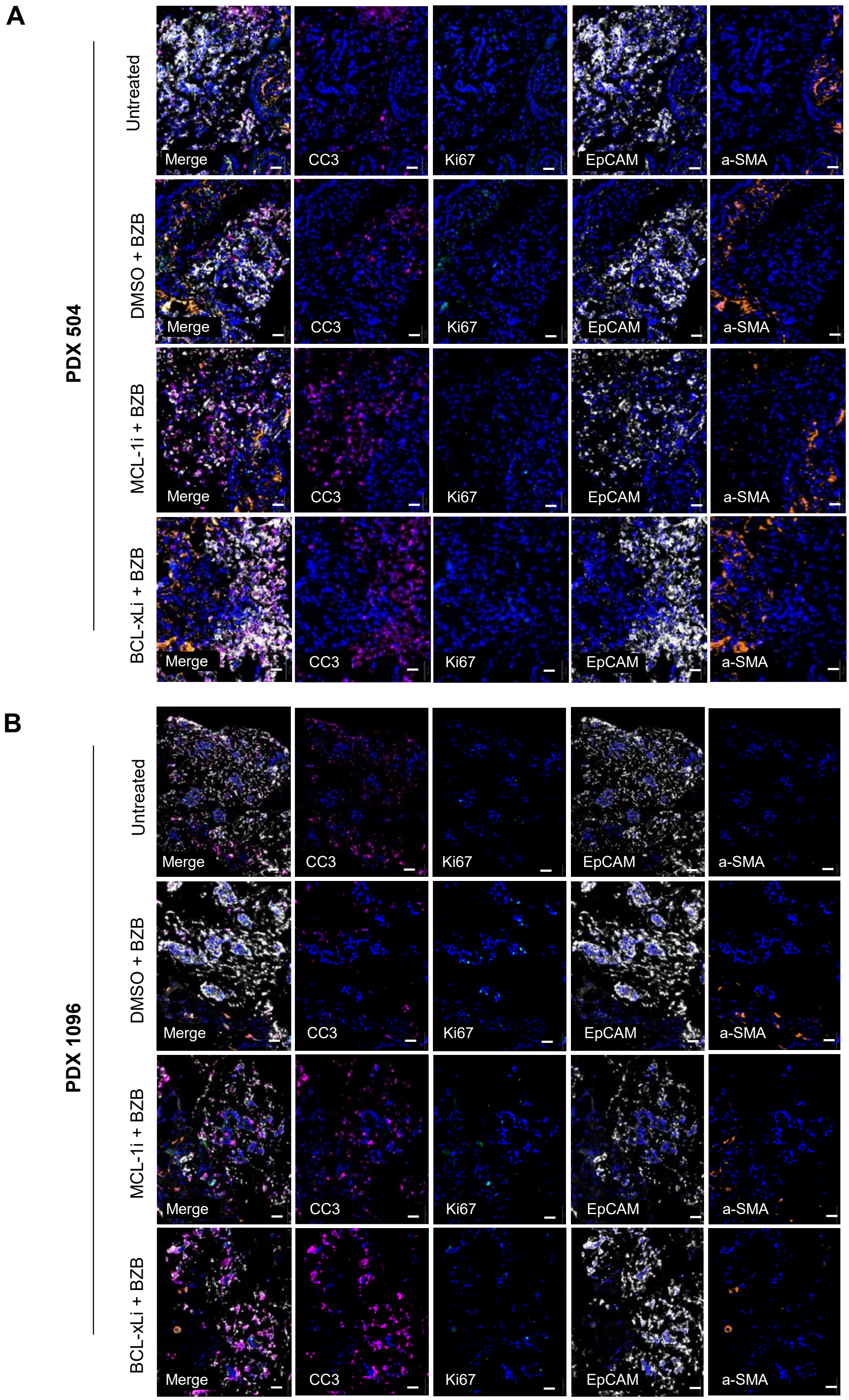
A
PDX 504
Untreated
DMSO + BZB
MCL-1i + BZB
BCL-xLi + BZB
Merge
CC3
Ki67
EpCAM
a-SMA
B
PDX 1096
Untreated
DMSO + BZB
MCL-1i + BZB
BCL-xLi + BZB
Merge
CC3
Ki67
EpCAM
a-SMA

Figure S12.

Figure S12. Tissue slices derived from cetuximab-resistant, KRAS wild-type PDX tumors are sensitive towards cell death-inducing agents. (A, B) Tissue slices were treated with 10 µM BH3-mimetic drugs targeting MCL-1i or BCL-xLi in the presence of 5 nM bortezomib for 24 to 48 hours. Untreated tissue slices were cultivated in parallel to monitor the overall viability and morphology. The 3 µM paraffin sections of tissue slices were derived from the models (A) PDX 504 and (B) PDX 1096, which were stained for cleaved caspase 3 (CC3; pink), Ki67 (green), EpCAM (white), a-SMA (orange), and DAPI (blue). Scale bars represent 50 µm. BZB, bortezomib; a-SMA, alpha-smooth muscle actin.
