# Supplementary material for: BCL-xL as a therapeutic target in cetuximab-refractory colorectal cancer
Source: Cell Death Dis. 2026 Jan 31;17(1):187. doi: 10.1038/s41419-026-08434-5 (PMC12876907; doi:10.1038/s41419-026-08434-5)
Supplement: Supplementary file 2 — Supplemental Tables [file 41419_2026_8434_MOESM2_ESM.pdf]

Table S1.

Table S1. Mutational profile of parental and cetuximab-resistant LIM1215 cell lines. Oncogenic mutations in the LIM1215 cell lines were identified by RNA sequencing analysis using the Integrated Genome Viewer. Par, parental; rep 1-3, replicate 1-3; wt, wild-type.

Gene, exon	Par_Rep 1	Par_Rep 2	Par_Rep 3	R1_Rep 1	R1_Rep 2	R1_Rep 3	R2_Rep 1	R2_Rep 2	R2_Rep 3
KRAS, exon 2	wt	wt	wt	wt	wt	wt	wt	wt	wt
KRAS, exon 3	wt	wt	wt	wt	wt	wt	wt	wt	wt
KRAS, exon 4	wt	wt	wt	wt	wt	wt	wt	wt	wt
BRAF, exon 11	wt	wt	wt	wt	wt	wt	wt	wt	wt
BRAF, exon 15	wt	wt	wt	wt	wt	wt	wt	wt	wt
PTEN, exon 7	wt	wt	wt	wt	wt	wt	wt	wt	wt
PTEN, exon 8	wt	wt	wt	wt	wt	wt	wt	wt	wt
EGFR, exon 12	wt	wt	wt	wt	wt	wt	wt	wt	wt
EGFR, exon 20	wt	wt	wt	wt	wt	wt	wt	wt	wt
NRAS, exon 2	wt	wt	wt	wt	wt	wt	wt	wt	wt
NRAS, exon 3	wt	wt	wt	wt	wt	wt	wt	wt	wt
NRAS, exon 4	wt	wt	wt	wt	wt	wt	wt	wt	wt
HRAS, exon 2	wt	wt	wt	G12D	G12D	G12D	wt	wt	wt
HRAS, exon 3	wt	wt	wt	wt	wt	wt	Q61H	Q61H	Q61H
HRAS, exon 4	wt	wt	wt	wt	wt	wt	wt	wt	wt
PIK3CA, exon 9	wt	wt	wt	wt	wt	wt	wt	wt	wt
PIK3CA, exon 20	wt	wt	wt	wt	wt	wt	wt	wt	wt
TP53, exon 5	wt	wt	wt	wt	wt	wt	wt	wt	wt
TP53, exon 7	wt	wt	wt	wt	wt	wt	wt	wt	wt
TP53, exon 8	wt	wt	wt	wt	wt	wt	wt	wt	wt

Table S2.

Table S2. Differentially expressed genes in LIM1215-R1 vs. LIM1215-parental cells. The gene expression of major apoptotic regulators in LIM1215-R1 cells was determined by RNA sequencing analysis, using the LIM1215-parental cells as a reference to determine differentially expressed genes. The selection of apoptosis-related genes is based on the KEGG apoptosis pathway (hsa04210).

Gene	log2FC	pvalue	padj
APAF1	-0.210	1.03E-02	2.72E-02
BAD	Exclusion during pre-filtering in the RNA-seq pipeline		
BAK	Exclusion during pre-filtering in the RNA-seq pipeline		
BAX	0.432	1.45E-08	1.20E-07
BBC3 (PUMA)	Exclusion during pre-filtering in the RNA-seq pipeline		
BCL-2	1.178	3.95E-07	2.66E-06
BCL2L1 (BCL-xL)	0.358	4.13E-12	5.00E-11
BCL2L11 (BIM)	Exclusion during pre-filtering in the RNA-seq pipeline		
BID	0.282	1.45E-04	6.04E-04
CASP3	Exclusion during pre-filtering in the RNA-seq pipeline		
CASP7	Exclusion during pre-filtering in the RNA-seq pipeline		
CASP9	Exclusion during pre-filtering in the RNA-seq pipeline		
DIABLO (Smac)	Exclusion during pre-filtering in the RNA-seq pipeline		
FADD	0.251	2.67E-04	1.05E-03
FAS	1.038	8.52E-29	2.80E-27
MCL-1	0.569	6.15E-29	2.03E-27
NOXA	Exclusion during pre-filtering in the RNA-seq pipeline		
TNFRSF10A	0.439	3.84E-11	4.25E-10
TNFRSF10B	0.521	4.11E-21	9.32E-20
XIAP	Exclusion during pre-filtering in the RNA-seq pipeline		

Table S3.

Table S3. Differentially expressed genes in LIM1215-R2 vs. LIM1215-parental cells. The gene expression of major apoptotic regulators in LIM1215-R2 cells was determined by RNA sequencing analysis, using the LIM1215-parental cells as a reference to determine differentially expressed genes. The selection of apoptosis-related genes is based on the KEGG apoptosis pathway (hsa04210).

Gene	log2FC	pvalue	padj
APAF1	-0.241	2.79E-03	9.64E-03
BAD	Exclusion during pre-filtering in the RNA-seq pipeline		
BAK	Exclusion during pre-filtering in the RNA-seq pipeline		
BAX	Exclusion during pre-filtering in the RNA-seq pipeline		
BBC3 (Puma)	-0.408	2.58E-04	1.14E-03
BCL-2	1.186	4.06E-07	2.88E-06
BCL2L1 (BCL-xL)	0.361	2.56E-12	3.13E-11
BCL2L11 (BIM)	Exclusion during pre-filtering in the RNA-seq pipeline		
BID	0.142	3.99E-02	9.39E-02
CASP3	-0.133	1.27E-02	3.57E-02
CASP7	Exclusion during pre-filtering in the RNA-seq pipeline		
CASP9	Exclusion during pre-filtering in the RNA-seq pipeline		
DIABLO (Smac)	Exclusion during pre-filtering in the RNA-seq pipeline		
FADD	0.154	1.84E-02	4.90E-02
FAS	0.462	2.18E-07	1.60E-06
MCL-1	0.593	3.58E-31	1.29E-29
NOXA	Exclusion during pre-filtering in the RNA-seq pipeline		
TNFRSF10A	0.452	1.07E-11	1.24E-10
TNFRSF10B	0.279	1.85E-07	1.37E-06
XIAP	0.152	2.29E-02	5.91E-02

Table S4.

Table S4. Comprehensive PDX model data, including patient information and treatment details.

Model	Histology	Patient gender	Patient age	Ethnicity	Site of origin	Patient tumor differentiation	Stage at implantation	Prior therapy	PDX vascularization	PDX stroma content
504	Adeno carcinoma	Female	70	Caucasian	Colon	Poor	Duke B, N1	no	intermediate	7.0%
533	Adeno carcinoma	Male	38	Caucasian	Liver	Poor	M1 liver	FU	high	10.0%
742	Adeno carcinoma	Male	66	Caucasian	Liver	Poor	M1 mesenterium	not known	high	15.0%
1096	Adeno carcinoma	Male	69	Caucasian	Skin	Not Known	M1 skin	not known	intermediate	6.7%

Table S5.

Table S5. Mutational profile of the cetuximab-resistant, KRAS wild-type PDX models 504, 533, 742 and 1096 by whole exome sequencing. wt, wild-type.

Gene	PDX 504	PDX 533	PDX 742	PDX 1096
BRAF	V600E	N49KIIKI	V600E	wt
EGFR	wt	wt	wt	wt
HRAS	wt	wt	wt	wt
KRAS	wt	wt	wt	wt
NRAS	wt	wt	wt	wt
PIK3CA	wt	wt	wt	wt
PTEN	wt	undefined	wt	gene deletion
T53	R282W	R175H	R273C	R175H
