# Supplementary material for: BCL-xL as a therapeutic target in cetuximab-refractory colorectal cancer
Source: Cell Death Dis. 2026 Jan 31;17(1):187. doi: 10.1038/s41419-026-08434-5 (PMC12876907; doi:10.1038/s41419-026-08434-5)
Supplement: Supplementary file 3 — Supplemental material and methods [file 41419_2026_8434_MOESM3_ESM.docx]

# **Supplementary Material and Methods**

**Antibodies**

For flow cytometry, FITC-conjugated anti-mouse immunoglobulins antibody was purchased from Agilent (polyclonal goat anti-mouse immunoglobulins/FITC, goat F(ab')2, #F047902-2, Agilent, Waldbronn, Germany), anti-human HER2 antibody (24D2), anti-human HER3 (1B4C3), mouse IgG1 κ isotype control and mouse IgG2a κ isotype control were purchased from BioLegend (purified anti-human CD340 (erbB2/HER-2) antibody, #324402 [RRID:AB_756118]; purified anti-human erbB3/HER-3 Antibody, #324702 [RRID:AB_756156]; purified mouse IgG1, κ Isotype ctrl antibody, clone: MOPC-21, #400102 [RRID:AB_2891079]; purified mouse IgG2a, κ Isotype ctrl antibody, clone: MOPC-173, #400202 [RRID: AB_2927399], BioLegend, Fell, Germany). For cellular ATP assays, anti-human panitumumab, IgG hu225, IgG 3-43 and scDb hu225x3-43-Fc were produced in-house and described before (1, 2) and trastuzumab was provided by Roche Diagnostics (Basel, Switzerland). For immunoblot analysis, antibodies were purchased from Cell Signaling (BID rabbit mAb #2002 [RRID:AB_10692485]; Bim (C34C5) rabbit mAb #2933 [RRID:AB_1030947]; Bak rabbit pAb #3814 [RRID:AB_2290287]; Ras (G12D Mutant Specific) (D8H7) rabbit mAb #14429 [RRID:AB_2728748]; MCL-1 rabbit mAb (D35A5) #5453 [RRID:AB_10694494]; PARP rabbit pAb #9542 [RRID:AB_2160739], Cell Signaling Technology Europe B.V; Frankfurt am Main, Germany), from Dianova (goat IgG anti-mouse IgG (H+L)-HRPO, MinX Hu,Bo,Ho pAb #115-035-062 [RRID:AB_2338504]; goat IgG anti-rabbit (H+L)-HRPO, MinX Hu,Ms,Rt pAb #111-035-114 [RRID:AB_2307391], Hamburg, Germany), from OriGene (KRAS, clone OTI2C1, mouse mAb #TA801672 [RRID:AB_2626032], OriGene Technologies Inc., Rockville, MD, USA), from Santa Cruz (Neu/ErbB-2 rabbit pAb #sc-284 [RRID:AB_632013]; ErbB-3, clone: C-17, rabbit pAb #sc-285 [RRID:AB_2099723], Santa Cruz Biotechnology, TX, USA), from Sigma-Aldrich (Actin mouse mAb #A4700 [RRID:AB_476730], Vinculin mouse mAb #V9131, [RRID:AB_477629], Taufkirchen, Germany), and from Proteintech (HRAS mouse pAb #18295-1-AP [RRID:AB_2121046], Proteintech Group, Illinois, USA).

**Mutational analysis**

DNA was extracted using the NucleoBond® Xtra Midi DNA isolation kit (Macherey-Nagel, Dueren, Germany) and sanger sequencing was carried out by Microsynth AG (Balgach, Switzerland) using standard protocols. Primer sequences for KRAS exon 2 and 3 were described in (3).

**Gene copy number analysis**

Parental and resistant cells were washed with PBS and harvested, followed by DNA extraction using the NucleoBond® Xtra Midi DNA isolation kit (Macherey-Nagel) according to the manufacturer’s instructions. Real-time PCR was performed using the Power SYBR® Green RNA-to-CT™ 1-Step Kit (Qiagen) by adapting the cycling protocol accordingly for the use of genomic DNA template with the CFX96 Touch (Bio-Rad). Gene copy number of resistant cells were compared to parental LIM1215 cells. Primers targeting the centromeric regions of chromosome 12 were used to normalize the data for aneuploidy. Primers for the gene copy number analysis were described in (3).

**Supplementary References**

1. Rau A, Lieb WS, Seifert O, Honer J, Birnstock D, Richter F et al. Inhibition of Tumor Cell Growth and Cancer Stem Cell Expansion by a Bispecific Antibody Targeting EGFR and HER3. Mol Cancer Ther 2020; 19(7):1474–85.

2. Schmitt LC, Rau A, Seifert O, Honer J, Hutt M, Schmid S et al. Inhibition of HER3 activation and tumor growth with a human antibody binding to a conserved epitope formed by domain III and IV. MAbs 2017; 9(5):831–43.

3. Misale S, Arena S, Lamba S, Siravegna G, Lallo A, Hobor S et al. Blockade of EGFR and MEK intercepts heterogeneous mechanisms of acquired resistance to anti-EGFR therapies in colorectal cancer. Sci Transl Med 2014; 6(224):224ra26.
