# Supplementary material for: BCL-xL as a therapeutic target in cetuximab-refractory colorectal cancer
Source: Cell Death Dis. 2026 Jan 31;17(1):187. doi: 10.1038/s41419-026-08434-5 (PMC12876907; doi:10.1038/s41419-026-08434-5)

Supplemental Material: Uncropped Western Blots

Figure 1E.

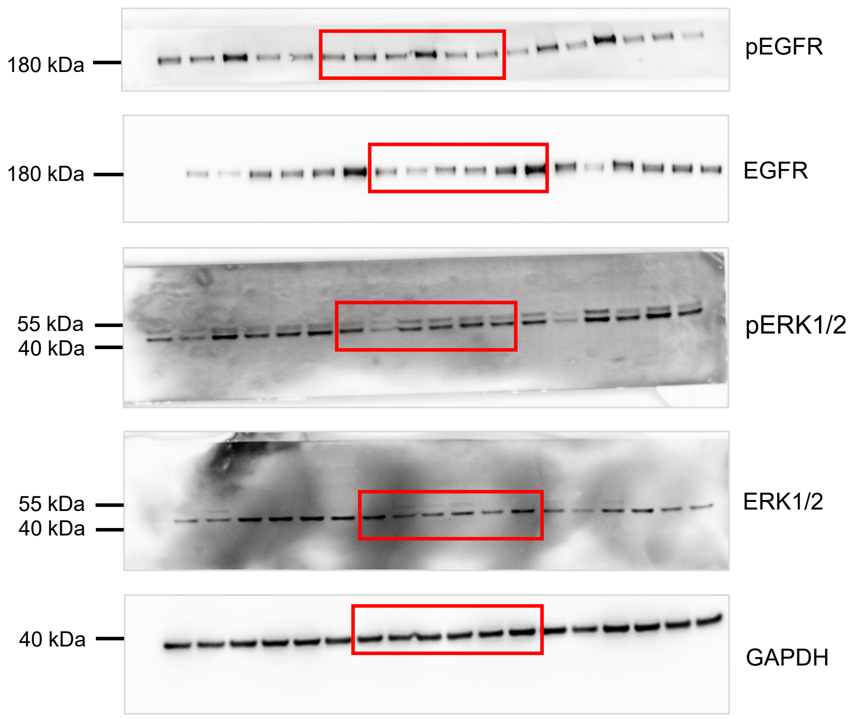

Supplemental Material: Uncropped Western Blots

Figure 2A.

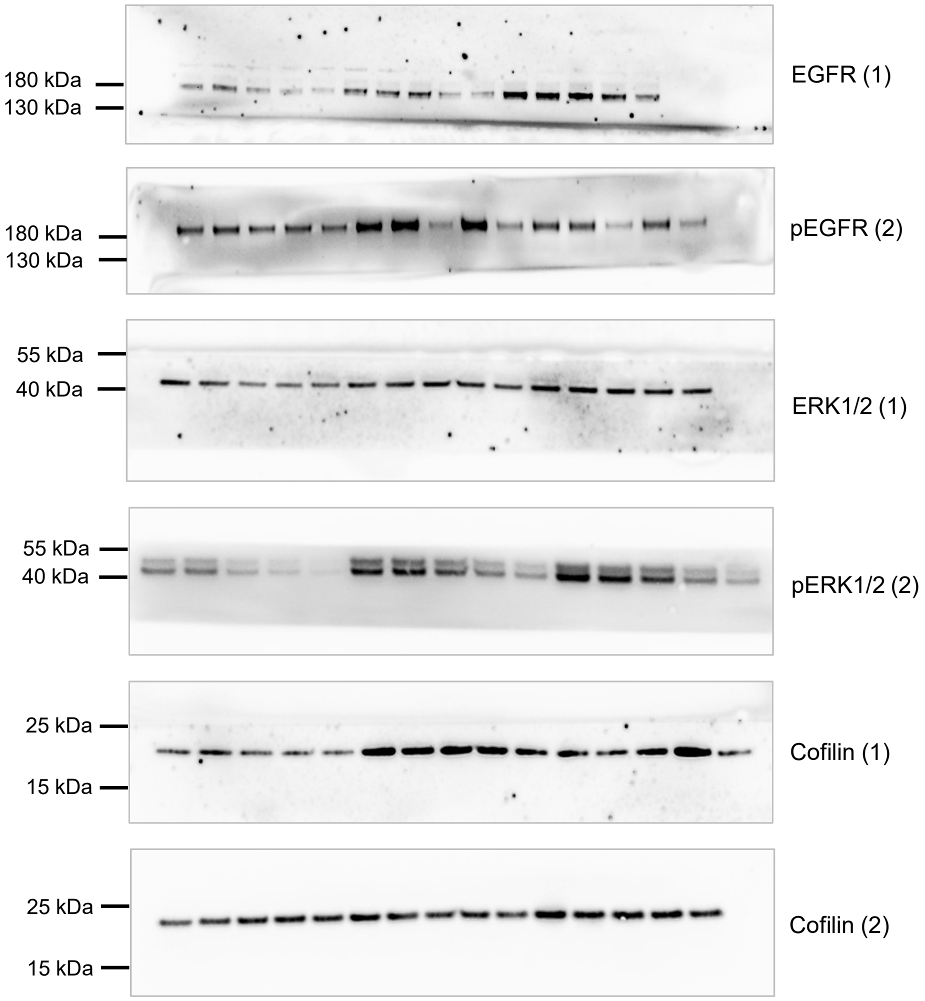

Supplemental Material: Uncropped Western Blots

Figure 3G.

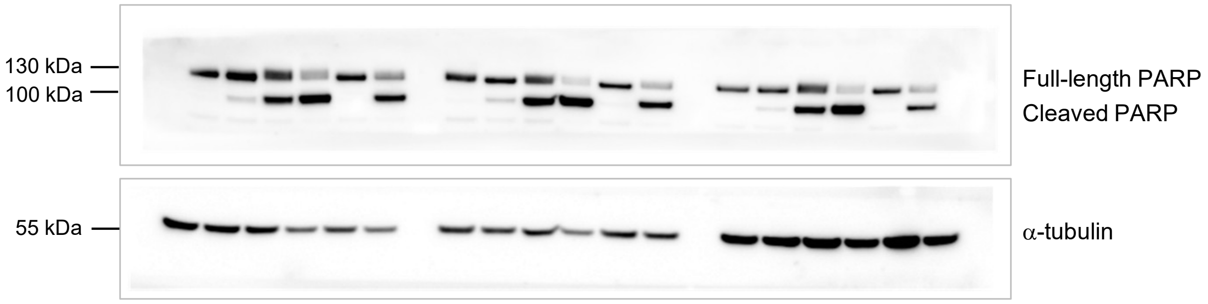

Supplemental Material: Uncropped Western Blots

Supplementary Figure S1C

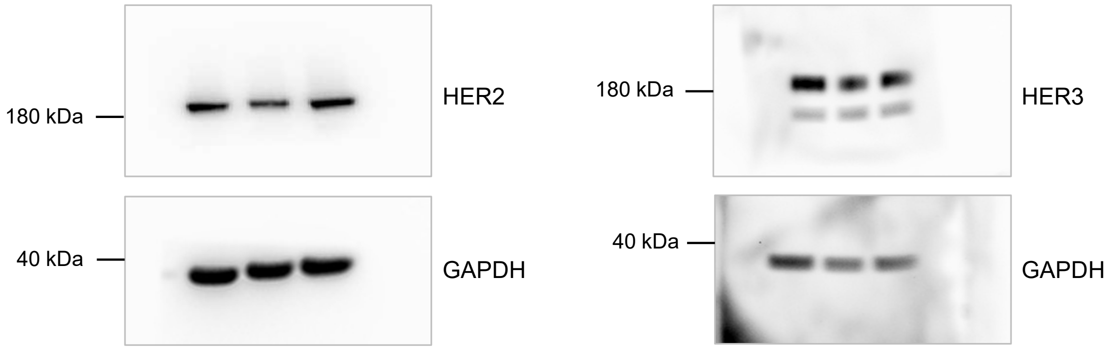

Supplementary Figure S2C

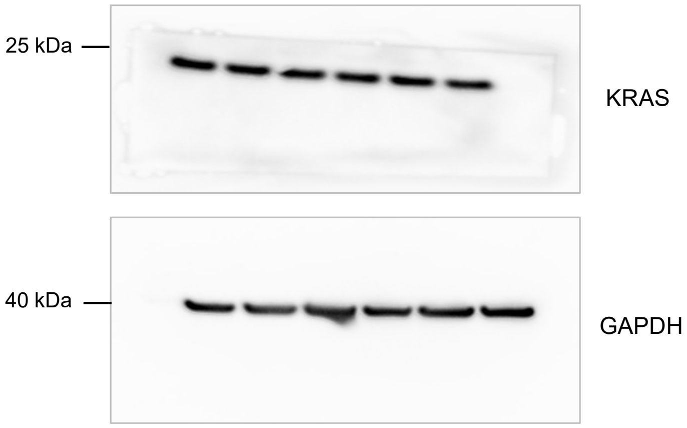

Supplemental Material: Uncropped Western Blots

Supplementary Figure S5.

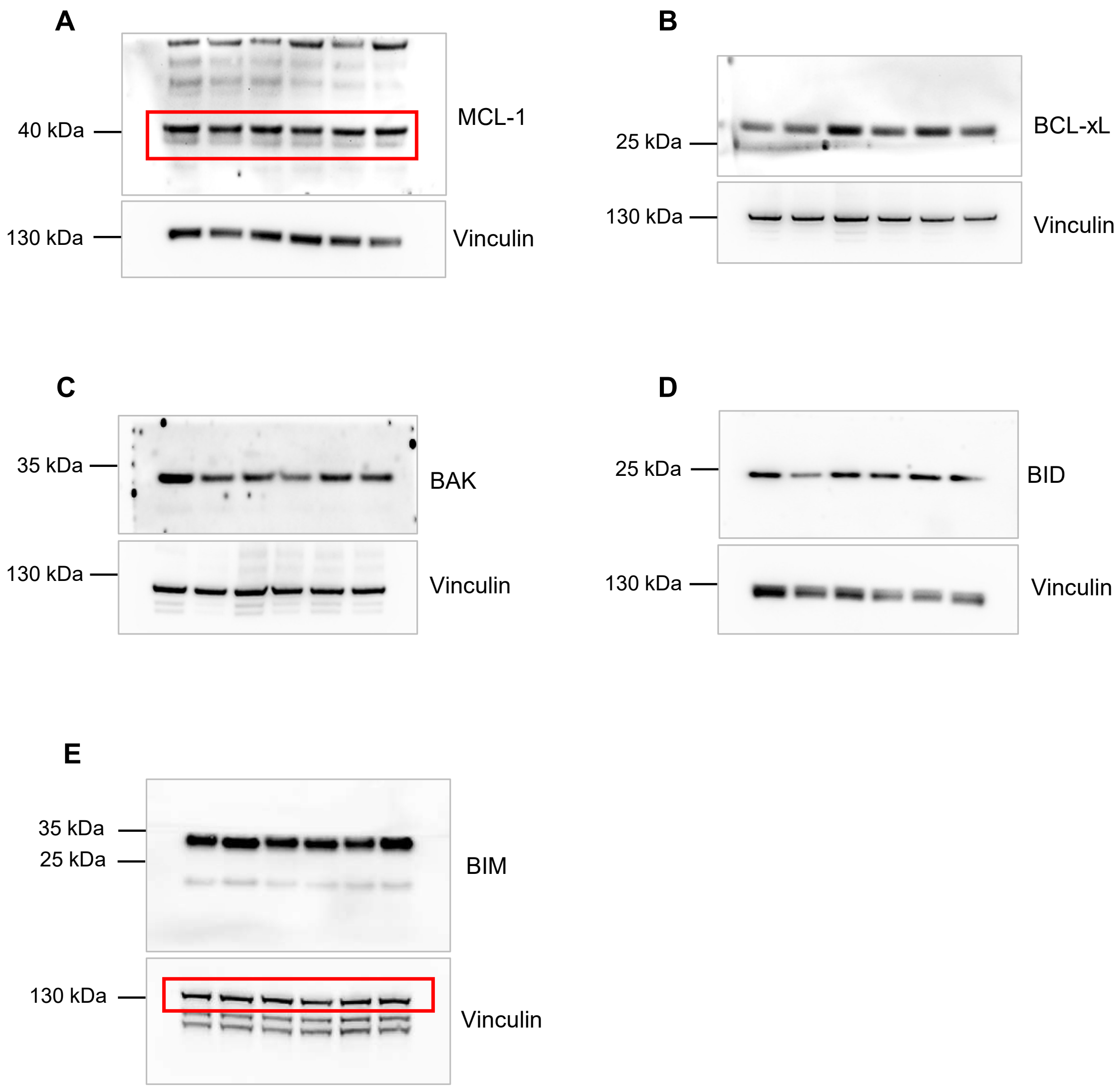

Supplemental Material: Uncropped Western Blots

Supplementary Figure S6.

A

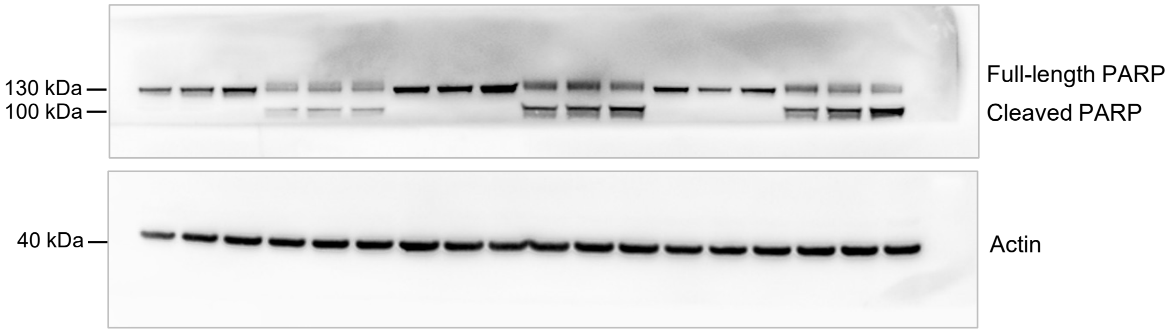

B

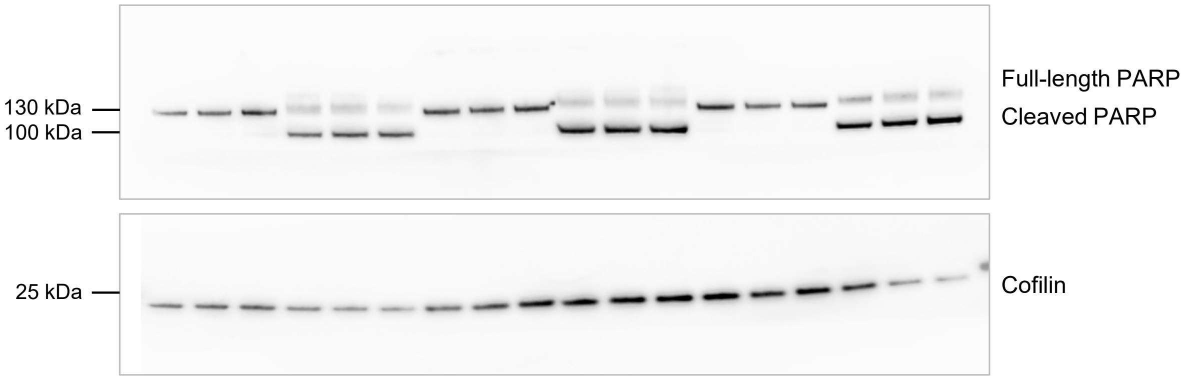

Supplemental Material: Uncropped Western Blots

Supplementary Figure S10.

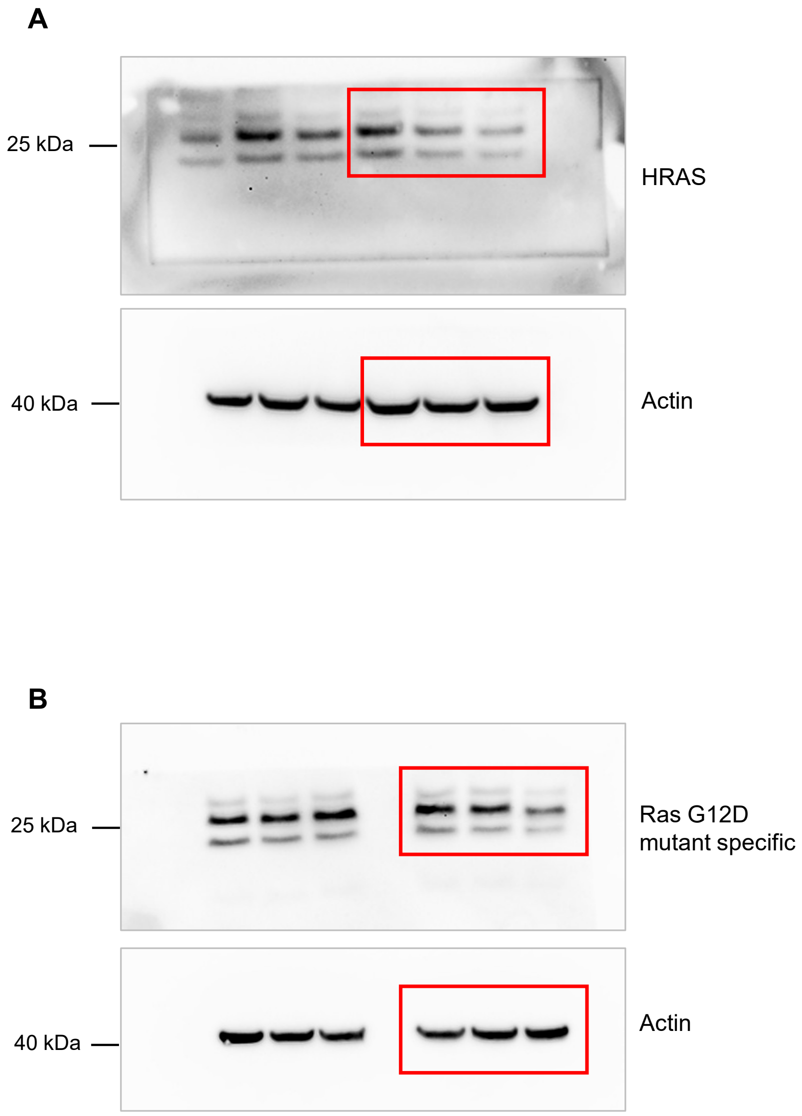

Supplemental Material: Uncropped Western Blots

Supplementary Figure S10.

C

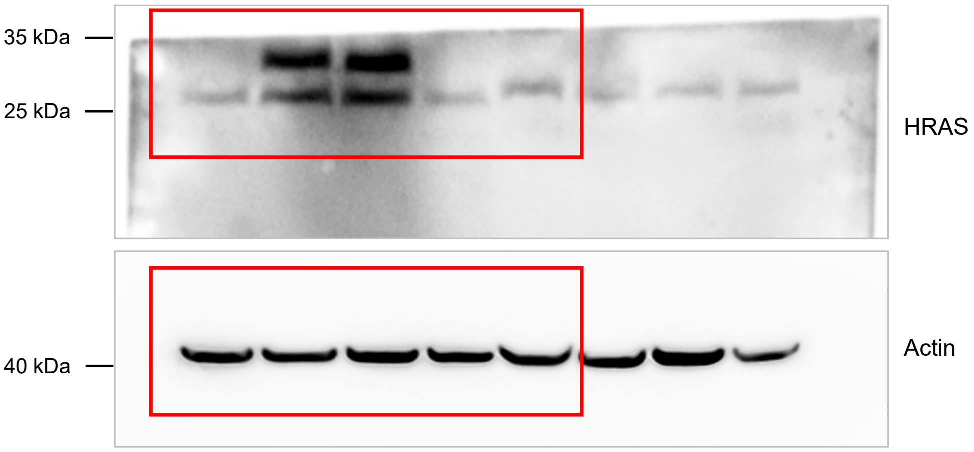

D

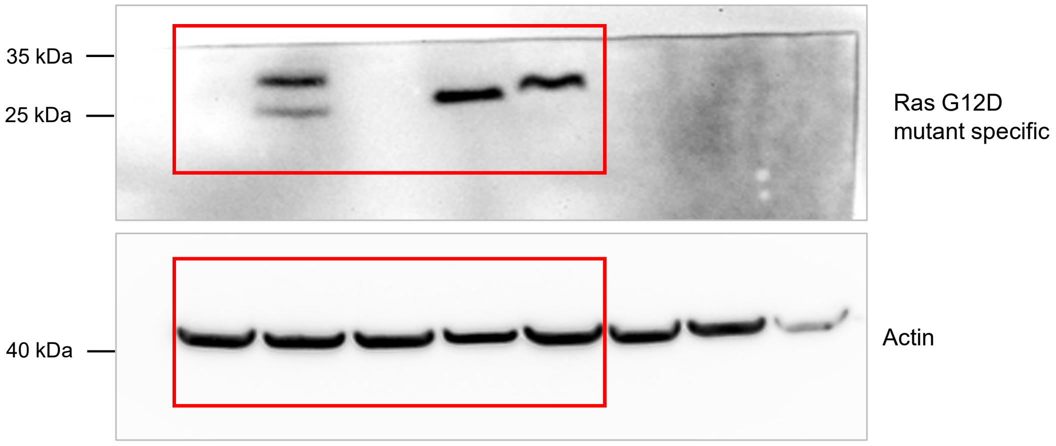

Supplement: Supplementary file 4 — Uncropped western blots [file 41419_2026_8434_MOESM4_ESM.pdf]
